# TDP-43 Epigenetic Facets and Their Neurodegenerative Implications

**DOI:** 10.3390/ijms241813807

**Published:** 2023-09-07

**Authors:** Juliette Gimenez, Alida Spalloni, Sara Cappelli, Francesca Ciaiola, Valerio Orlando, Emanuele Buratti, Patrizia Longone

**Affiliations:** 1 Molecular Neurobiology Laboratory, Experimental Neuroscience, IRCCS Fondazione Santa Lucia (FSL), 00143 Rome, Italy; a.spalloni@hsantalucia.it (A.S.); p.longone@hsantalucia.it (P.L.); 2 Molecular Pathology Laboratory, International Centre for Genetic Engineering and Biotechnology (ICGEB), 34149 Trieste, Italy; sara.cappelli@icgeb.org (S.C.); emanuele.buratti@icgeb.org (E.B.); 3 Department of Systems Medicine, University of Roma Tor Vergata, 00133 Rome, Italy; 4 KAUST Environmental Epigenetics Program, Biological Environmental Sciences and Engineering Division BESE, King Abdullah University of Science and Technology (KAUST), Thuwal 23955, Saudi Arabia; valerio.orlando@kaust.edu.sa

**Keywords:** TDP-43, TARDBP, neurodegeneration, ALS, FTD/FTLD, epigenetics, chromatin, transcriptional regulation, DNA repair, retrotransposon

## Abstract

Since its initial involvement in numerous neurodegenerative pathologies in 2006, either as a principal actor or as a cofactor, new pathologies implicating transactive response (TAR) DNA-binding protein 43 (TDP-43) are regularly emerging also beyond the neuronal system. This reflects the fact that TDP-43 functions are particularly complex and broad in a great variety of human cells. In neurodegenerative diseases, this protein is often pathologically delocalized to the cytoplasm, where it irreversibly aggregates and is subjected to various post-translational modifications such as phosphorylation, polyubiquitination, and cleavage. Until a few years ago, the research emphasis has been focused particularly on the impacts of this aggregation and/or on its widely described role in complex RNA splicing, whether related to loss- or gain-of-function mechanisms. Interestingly, recent studies have strengthened the knowledge of TDP-43 activity at the chromatin level and its implication in the regulation of DNA transcription and stability. These discoveries have highlighted new features regarding its own transcriptional regulation and suggested additional mechanistic and disease models for the effects of TPD-43. In this review, we aim to give a comprehensive view of the potential epigenetic (de)regulations driven by (and driving) this multitask DNA/RNA-binding protein.

## 1. Introduction

Epigenetic mechanisms in both general and specific neurodegenerative diseases are gaining momentum [1,2,3,4,5,6]. The present review is centered on the epigenetics of the transactive response (TAR) DNA-binding protein 43 (TDP-43) and its implications for neurodegenerative disorders.

TDP-43 gained momentum in the neurodegeneration field when it was first discovered that almost all amyotrophic lateral sclerosis (ALS) cases and as many as half of frontotemporal dementia (FTD) cases present pathological ubiquitinated inclusions of TDP-43 [7,8]. Since then, deregulated TDP-43 has been described in several neurodegenerative diseases with different degrees of penetration, from ALS (97%) to FTD (45–50%) to Alzheimer’s disease (AD, 40–50%) (as reviewed in [9]). TDP-43 aggregates have also been found in patients with Huntington’s disease [10,11], in the brains of humans following traumatic brain injury (TBI) [12,13], and in an increasing list of neurodegenerative or aging-related diseases [14]. More recently, alterations in TDP-43 regulation/aggregation have also been described in many patients affected by inclusion body myositis [15] and in models of Niemann–Pick disease [16,17], thus extending the list of TDP-43-associated diseases beyond the strict neurodegenerative spectrum.

At the functional level, TDP-43 is a highly conserved protein involved in the regulation of RNA processing. Mechanistically, TDP-43 is able to bind with different affinity both single-stranded (ss)RNA and ssDNA but also double-stranded DNA (dsDNA). From a structural point of view, the binding of TDP-43 to nucleic acids is mediated by two RNA recognition motif (RRM) domains in its N-terminal region [15,16,17,18,19,20,21,22,23].

In agreement with its strong affinity for nucleic acids, TDP-43 localization is mainly nuclear, and cytoplasmic aggregation is attributed to pathological processes. However, it is now well recognized that TDP-43 is capable of shuttling back and forth from the nucleus to the cytoplasm, even under normal physiological conditions [24]. On the one hand, in neurons, TDP-43 is able to shift to the cytoplasm and travel along the axons to bring mRNA to the synapses for local translation, a function notably impaired in stem-cell-derived motor neurons from ALS patients bearing TDP-43 ALS-causing mutations [25,26,27,28]. On the other hand, pathological TDP-43 accumulates in dense cytoplasmic inclusions that include full-length protein and protease cleavage products such as C-terminal TDP-43 fragments (CTFs), as well as abnormally phosphorylated and ubiquitinated proteins [9,29,30,31]. When irreversibly aggregated in the cytoplasm, it is believed that the protein is unable to perform its normal functions and thus leads to a loss-of-function scenario (although gain-of-function consequences may be present).

Being a DNA/RNA-binding protein, TDP-43 is highly involved in many aspects of RNA metabolism, such as the control of alternative splicing (AS), microRNA (miRNA) processing, and messenger RNA (mRNA) stability and transport [32,33,34,35,36]. Nonetheless, recent reports have broadened its function to the regulation of a wide range of chromatin features, from gene transcriptional regulation to DNA repair, passing by chromatin shaping, and the control of retrotransposons for DNA stability. However, because of the abundance of biological functions, it is still yet unclear which ones are early/central to neurodegenerative processes. In this review, we aim to give a general survey of these many epigenetic facets of TDP-43 that have arisen in recent research and of the epigenetic mechanisms leading to TDP-43 regulation. Finally, we also aim to provide specific highlights on the neurodegenerative diseases in which these processes are dysregulated.

## 2. TDP-43 Role in Chromatin Remodeling and Transcription

During the last decade, TDP-43 has been mostly studied for the functions linked to its RNA-binding properties. Notwithstanding this focus, initial studies established the capacity of TDP-43 to bind to ssDNA TG repeats with at least the same efficiency as it does to *UG*-repeated sequences within RNA [15,16,19,22], while binding to other motifs, e.g., to the ssDNA HIV TAR motif from which its name is derived, performed at a slower association rate and at an even slower dissociation rate than it does to (TG)6 stretch [19,20]. Instead, its ability to bind dsDNA has been documented in different studies [15,20,21] and notably regarding free dsDNA ends [23].

More recently, additional studies have found TDP-43 to be able to specifically bind DNA sequences in promoter regions and affect the expression of several genes [15,19,37,38,39,40,41,42,43,44,45,46,47,48,49], discussed in detail below. Although the function of TDP-43 on chromatin is yet to be fully understood, it is now very clear that the toxic effects of altered TDP-43 can affect chromatin homeostasis.

### 2.1. TDP-43 Is a Global Chromatin Modifier

As already mentioned, the function of TDP-43 as a more general transcriptional activator/repressor has been known for about a decade [40]. Its involvement in chromatin silencing and nuclear/cytoplasmic shuttling constitute convergent key findings from several biological screens, and several crucial epigenetic factors appear to be able to modify TDP-43-induced degeneration [50,51,52,53].

For example, using a mosaic genetic screen to study motor neuron degeneration in the *Drosophila* leg, Sreedharan et al. identified three factors, namely, *sgg/GSK3*, *hat-trick*, and *xmas-2*, needed to mediate TDP-43^Q331K^ toxicity [50]. Interestingly, they noted that the manipulation of these three modifiers did not rescue Wallerian degeneration, another neurodegenerative but TDP-43-independent disease [50]. Among these three proteins, Shaggy (sgg), probably a downstream target of TDP-43, suppressed TDP-43 toxicity without reducing its expression [50]. In parallel, previous screening studies supported a mechanistic link between TDP-43 and Glycogen Synthase Kinase 3 (GSK3). They reported that TDP-43 activates GSK3, while GSK3 inhibition reduces TDP-43 aggregation [54,55]. Finally, the loss of the two other factors, *xmas-2* or *hat-trick*, implicated in chromatin remodeling and RNA export, affected TDP-43 post-transcriptionally, resulting in a reduction in TDP-43 protein level [50].

In addition to this evidence, two recent studies using the suppressor screen techniques have led to the identifications of other epigenetic modifiers able to contrast TDP-43 neurotoxicity. In these two studies, human TDP-43 was over-expressed in a subset of *Drosophila* photoreceptor neurons [51], motor neurons, or glial cells [52]. Then, using a combination of shRNA or CRISPR/Cas9 knockdown (KD) screens, these cells were used to identify suppressors of TDP-43 neurotoxicity. Specifically, Azpurua and colleagues used an age-dependent neurobehavioral defect as a primary readout [52], while Berson and colleagues used the red eye degeneration readout [51].

Numerous genes implicated in nucleocytoplasmic transport or in pathways that are deregulated in TDP-43-related neurodegeneration were identified among the glial and motoneuronal TDP-43 suppressors of toxicity. However TDP-43-phenotype suppressors were principally composed of chromatin remodeling and basal transcription machinery factors. In particular, 25% of them were chromatin remodelers. Seven out of eight of these factors promote open chromatin as part of the Trithorax and SWI/SNF (Brahma) complexes, most of them with human known homologues: *e*(*y*)*3/PHF10*; *polybromo/BAF180*; *ash1/ASH1L*; *enok/KAT6A*; *br/-*; *Br140/BRPF1*; and *mor/BAF170S/MARCC2*. The remaining, the Chromodomain-helicase-DNA binding protein 1 (Chd1) with two human homologues, CHD1/CHD2, is an ATPase involved in the remodeling and assembly of chromatin [52]. Further analyses showed that TDP-43 can physically interact with fly Chd1 and human CHD2, impeding their recruitment onto chromatin. Interestingly, both proteins were clearly observed in the chromatin fractions but the Chd1-TDP-43 interaction did not take place on chromatin; rather, it was specifically observed in the cell-soluble fractions both in Drosophila and in human HEK293 cells [55]. By hijacking Chd1, overexpression of TDP-43 resulted in the impairment of correct nucleosome clearing from the gene body of a specific set of stress-protecting genes, preventing their activation [51]. The Chd1-TDP-43 interaction axis might therefore be one way by which the upregulation of TDP-43 sensitizes cells to various stress. 

Importantly, according to the different brain cells investigated, it was also observed that *Chd1* KD could cause an opposite effect on TDP-43 overexpression-mediated toxicity; in particular, instead of counteracting the effects of TDP-43 overexpression upon motor neurons KD, an exacerbation of toxicity was observed upon glial and photoreceptor neurons KD [51,52]. These data highlight the importance of the cellular context for mediating TDP-43 activity, an important parameter that has been recently observed for TDP-43 pre-mRNA splicing regulatory properties as well [56].

Changing the model organism to *C. elegans,* it was found that the TDP-43 homolog, TDP-1, could regulate the chromatin localization of another chromatin remodeler, HPL-2, the heterochromatin protein 1 homolog [53]. Direct interaction was found to occur between these two proteins, both in the presence and absence of RNA. In this study, it was shown that TDP-1 facilitates HPL-2 association with active genes to maintain mRNA abundance. In addition, chromatin immunoprecipitation (ChIP) experiments indicated that TDP-1 is present at most of the HPL-2 peaks on chromatin in this organism. Specifically, loss of TDP-1 decreased most of the HPL-2 peaks where (AC)n and (AG)n binding motifs were present. These regions were located predominantly in intronic regions (71%) and promoters (20%) with levels of corresponding RNA decreasing in a *tdp-1* mutant worm [53]. As a side note, the intronic localization of TDP-1 on DNA could be related to the propensity of TDP-1/TDP-43 orthologs to bind pre-mRNA chiefly within introns, as previously demonstrated in multiple organisms [34,35]. At the genome-wide level instead, TDP-43 was found to enrich particularly at promoter regulatory regions, as we will review in the next sections. 

Additional evidence of TDP-43 function in chromatin remodeling and its relevance to neurodegenerative diseases comes from the study of nBAFs proteins in cultured mouse motor neurons expressing ALS-linked mutant (G418C and A315T) human TDP-43 [57]. The Brahma-related gene 1 (Brg1)-associated factor (nBAF) chromatin-remodeling complex is critical for neuronal differentiation, dendritic extension, and synaptic function. In this study, the authors showed that nBAF subunits were lost in cultured mouse motor neurons expressing both mutants of human TDP-43. The decrease in nuclear Brg1, BAF53b, and CREST was observed when either mutant was expressed, but also when WT human TDP-43 protein expression was shifted to neuronal cytoplasmic inclusions, thus suggesting TDP-43 as a positive regulator of nBAF expression. In agreement with this conclusion, when co-expressed with mutant TDP-43, the presence of Brg1 delayed the induced dendritic attrition [57]. These data indicate that nuclear loss of TDP-43 can lead to a decrease in nBAF subunits production, either because of a transcriptional repression mechanism or following a defect in RNA processing, potentially leading to RNA nuclear retention, such as the one observed for Brg1 mRNA [57]. Nonetheless, it was interesting to observe that the depletion of nBAF subunits and the delayed attrition upon Brg1 co-expression were not unique to TDP-43; indeed, they were observed also for ALS-linked FUS mutants, and loss of nBAF subunits has also been reported to occur in spinal motor neurons of familial ALS (fALS) and sporadic ALS (sALS) patients with *C9orf72* GC expansion (C9ALS) or sALS without mutations in common ALS-linked genes [57].

The contribution of TDP-43, and especially of its ALS-related mutants to more global epigenome alteration, was also recently tested in the human neuroblastoma SH-SY5Y cell line, together with other ALS-causative proteins, SOD1 and FUS [58]. In this work, the authors investigated four modifications on histone H3 tail associated with either transcriptional activation: (i) H3 serine 10 phosphorylation and lysine 14 acetylation (H3S10Ph-K14Ac); and (ii) H3 lysine 4 dimethylation (H3K4me2); or with transcriptional repression marks: (iii) H3 trimethylation of K9H3K9me3; and (iv) DNA methylation. Recombinant adenoviral expression of WT or ALS-related mutants of either TDP-43, SOD1 or FUS proteins all triggered a dose-dependent decrease in cell vitality. However, statistically significant differences in epigenetic marks were limited and specific to the TDP-43 genotype. In particular, a significant decrease in global H3S10Ph-K14Ac was observed for TDP-43^M337V^, whereas TDP-43^WT^ overexpression led to a significant increase in H3K9me3. On the contrary, no relevant global losses or gains of these epigenetic marks were observed for the TDP-43^A382T^ mutant [58].

In line with these findings, the fly Chd1-TDP-43 interaction study previously mentioned was part of a broad in vivo RNAi screen to search for TDP-43 toxicity modifiers [51]. This screen investigated a total of 84 genes related to various aspects of chromatin biology, including histone methyltransferases (HMTs), demethylases (HDMs), acetyltransferases (HATs), and deacetylases (HDACs), as well as associated factors, histones, and chromatin remodelers. [51]. In addition to Chd1, it allowed for the identification of an additional 4 ‘‘strong’’ and 27 ‘‘mild’’ modifiers, both enhancers and suppressors of TDP-43-mediated eye degeneration. Most of them converged on the conclusion that the TDP-43-mediated toxicity is associated to H3K4me3-linked aberrantly closed chromatin. The modulation of genes that alter other histone methylation marks (repressive H3K27, active-gene body H3K36, or H3K79) mostly had no effect on TDP-43 toxicity [51], with the exception of H3K9 HMT Su(var)3-9. The suppression in flies of Su(var)3-9—that is, the homolog of human SUV39H1—diminished TDP-43-induced toxicity [51]. These observations support the finding by Masala et al. of an aberrant increase in H3K9me3 modification upon ectopic TDP-43^WT^ expression [58]. Note that this effect was not reported for the suppression of G9a, the other well-known H3K9 HMT [51].

Finally, two HDACs, HDAC1 and HDAC6, have been shown to influence and to be influenced by TDP-43, respectively. Thus, it has been shown that the silencing of both HDAC1 in SH-SY5Y and its fly ortholog Rpd3 in Drosophila is able to mitigate the toxic effect induced by TDP-43 expression [48]. Notably, this effect is possibly due to a direct modification of TDP-43 acetylation and consequent cellular localization and functional modulations, notably upon stress (see Section 2.2: TDP-43 and Local/Specific Gene Transcriptional Regulation). In 2010, two peer-reviewed studies showed that TDP-43 was able to bind *HDAC6* mRNA, regulating both its mRNA and protein expression in neuronal and non-neuronal cell lines [59,60]. In one of these studies, Tibbetts’s group demonstrated that this interaction was also mediated by FUS/TLS, which was able to form protein complexes and to share overlapping HDAC6 binding sites with TDP-43 [60]. Conversely, HDAC6 was later shown to exert a deacetylation activity on TDP-43. Indeed, HDAC6 mediated the removal of TDP-43 acetylation at the residues Lys-145 and Lys-192, induced by the CPB acetyltransferase. This was found to decrease the cytoplasmic TDP-43 accumulation in otherwise normal cellular conditions [61]. On the contrary, the formation of TDP-43 aggregates that was induced in case of strong oxidative stress promoted by arsenite could not be deacetylated by HDAC6 despite its interaction with TDP-43, which, overall, contributed to the accumulation of mature aggregates of TDP-43 [61]. In 2020, the relationship between TDP-43 and HDAC6 was further analyzed by Lee and collaborators [62]. They found that the overexpression of HDAC6 in a *Drosophila* model of TDP-43 proteinopathy reduced the amount of insoluble poly-ubiquitinated proteins and ameliorated the lifespan and climbing defects associated with the overexpression of both TDP-43 and Ataxin-2 (ATXN2). These results indicated that HDAC6 could modulate, albeit in a non-enzymatic manner, the TDP-43 activity via the autophagy–lysosome pathway (ALP) [62]. 

At the level of gene expression, substantial alterations were observed in the cortices of transgenic mice expressing inducible WT or mutant hTDP-43 lacking the nuclear localization signal (tTA/TDPΔNLS). These alterations appeared even before the onset of significant gliosis and neuronal cell loss [63]. Despite both human TDP-43 transgenes downregulating the endogenous mTDP-43 (by the well-known phenomenon of TDP-43 autoregulation (see specific section)), the mutant lacking the nuclear localization signal showed the most profound changes in gene expression. Among the many processes that were altered in these mice, “DNA–protein complex assembly” pathway was particularly affected and harbored genes coding for major nucleosome proteins. Specifically, many histone variants (H2bp, H3d, H4a/H4b/H4c, and H4h) and several nucleosome assembly protein-1-like1 (NAP1L1) genes were found. While the histone variants were all upregulated, the *NAP1L1* genes, on the contrary, were all downregulated [63]. Although these data were obtained using microarray, further RNA-seq analyses on the same model confirmed the alteration in transcription-related pathways and histone transcript levels [64]. In particular, it was observed that Med20, an essential component of the transcription-regulating Mediator complex, and Usp49, a histone H2B deubiquitinase which regulates splicing, were differentially spliced. In parallel, the canonical Histone *Hist1h3* and *Hist1h4* mRNAs were aberrantly polyadenylated, while at least 10 out of 15 variant histones were slightly but significantly downregulated in the TDPΔNLS bigenic mice [64]. In particular, enhanced cytoplasmic expression of TDP-43 downregulated histone 3′ UTR processing genes, notably Snrpe and Snrpd3, and a similar trend was observed for Lsm1l [64], thus further sustaining a role for TDP-43 in histone transcripts regulation.

To relate these findings to the human pathological condition, it is now known that not all cells in the brain of a patient present a reduced load of nuclear TDP-43, and the transcriptome of these cellular populations was recently investigated [65]. To achieve this, Liu et al. successfully separated diseased neuronal nuclei without TDP-43 from nuclei retaining nuclear TDP-43 in a post-mortem FTD and FTD–ALS human brain by combining subcellular fractionation and fluorescent-activated cell sorting (FACS) [65]. Subsequent transcriptome analysis has revealed abundant changes in gene expression associated with loss of TDP-43. In keeping with results obtained from the various animal models, the data from this human material confirmed that many altered genes were involved in histone processing. Furthermore, DNA damage and repair genes were found enriched in addition to genes affecting proteostasis, RNA processing, and nucleocytoplasmic transport. In particular, it was noted that a cluster of 10 altered genes, namely, HUWE1, YY1, MORF4L2, HMGN1, PRKDC, UIMC1, POLB, SFPQ, MSH3, and XRCC5/Ku70, were part of a DNA repair module [65].

DNA methylation is another major epigenetic modification, acting on DNA itself, rather than on the chromatin or nucleosomal proteins wrapped around it. At the biological level, DNA methylation is established via DNA methyltransferases (DNMTs) and is passively erased during DNA replication or, as can be more relevant for neuronal cells, by active replication-independent mechanisms involving oxidations steps mediated by the ten-eleven translocation (TET) enzymes and base excision repair [66,67,68]. DNA methylation in mammals mostly takes place at cytosines (5mC) in the cytosine–guanine dinucleotide context (CpG), but 5mCpH (CpA, CpT, CpC) are also found in the adult mammalian brain [69]. The majority of the CpG are methylated in mammals, with dense CpG islands often unmethylated. CpG islands generally lie in the genes’ regulatory regions and impact transcription. CpG methylation generally has a repressive function, notably controlling promoter activation, but it can also regulate splicing and DNA stability [66,67,70,71]. On the other hand, its first oxidized state, the hydroxymethylated C (5hmC), positively influences gene expression, notably in the human brain [72].

No relevant changes in global DNA methylation were observed by Masala et al. in the human neuroblastoma SH-SY5Y cell line overexpressing WT or mutant ALS-linked proteins, including TDP-43, as cited above [58]; however, the brains of ALS patients show a different trend; in fact, altered DNA methylation has been recently observed to occur in human post-mortem CNS tissues from ALS patients using immunohistochemistry. It consisted of higher levels of 5mC and h5mC in the residual lower motor neurons of both sALS and C9ALS compared to the same region in controls [73]. A significantly lower number of neurons with detectable 5mC (mean about 28% vs. >73%) and 5hmC (mean about 51% vs. >87%) was found among neuronal subpopulations with pathological nuclear TDP-43 loss (10% of neurons) compared to those with normal nuclear TDP-43, therefore linking TPD-43 nuclear loss to loss of DNA methylation (despite the direction of causation remaining unknown). Overall, these findings could be connected to differential DNA methylation of several hundreds of genes in ALS spinal cord motor neurons, mostly involved in RNA processing and splicing [73]. Very recently, Catanese and colleagues used multi-omics and machine learning to question the transcriptional, epigenetic, and mutational aspects of heterogeneous human IPSCs-derived motor neurons holding mutants of either *C9orf72*, *TARDBP*, *SOD1*, or *FUS*, as well as datasets from patients’ biopsies [74]. Analysis of both transcriptome and methylation data resulted in different patterns characterizing the different ALS mutations. Thus, several thousands of DMRs were identified in the ALS sub-group as compared to control, yet a fraction (123 hypermethylated, 179 hypomethylated DMRs) was common to all subgroups, and partially overlapped with the TARDBP mutations (G298S and N390D)-holding subgroup [74]. These results also highlight a deep heterogeneity within the different ALS subtypes on the epigenetic level. Analysis of the DMR-related biological processes, however, indicated that epigenetic abnormalities among ALS iPSCs MNs all contribute to the synaptic alterations (downregulations) observed in all the related transcriptomes, although different sets of synaptic genes were hinted depending on the ALS-related mutation. Nonetheless, all the ALS iPCS-derived MNs displayed upregulation of acetylcholine receptor-binding genes in conjunction with a hypo-methylation of their promoters, notably LY6E, LY6H, and PSCA [74]. In addition, proteomic analysis of proteins co-purifying with TDP-43 in mice brain nuclear extracts has previously identified methyl CpG-binding protein 2 (MeCP2) as an interactor of TDP-43 [75]. MeCP2 is a protein whose defects are responsible for the degenerative Rett Syndrome pathology that binds mC and hmC not only in the CpG context. Interestingly, MeCP2 appears to be implicated in several regulatory contexts similar to TDP-43 (genes and TE transcription and RNA splicing, chromatin loop organization, and heterochromatin structure) [76].

Cell cycle alterations have also been reported following TDP-43 suppression. In two recent publications, TDP-43 activity was linked to sister chromatid cohesion through the splicing regulation of a cohesin complex subunit, namely, Stromal Antigen 2 (STAG2). In particular, depletion of TDP-43 in HeLa and neuroblastoma cell lines upregulated STAG2 exon 30b inclusion [77,78]. According to those data, cell accumulation was observed in G2/S phase, further supporting the role of TDP-43 in multiple processes involving genome remodeling.

Finally, genes related to transcriptional machinery constitute another broad category of TDP-43-phenotype suppressors that have been identified thanks to several screening techniques. These genes include the transcription elongation factor, Su(Tpl) [52,79], which aberrantly expresses small nucleolar RNAs in TDP-43 pathology [79], *TAF1*, and *e*(*y*)*1* orthologs of the mammalian *TAF1* and *TAF9* transcription factors, members of the TFIID initiation complex, and also *Tombola* involved in the transcriptional activation of the male germline during meiosis [79]. Specifically, no less than eight genes coding for subunits of the Mediator (Med) complex, mediating RNA-polymerase interaction with transcription factors, were identified by Azpurua et al. [52]. The alteration of another Med subunit, Med20, was identified in mice cortices upon TPD-43 manipulations [64], thus reinforcing a potential role for TDP-43 in gene transcriptional regulation on chromatin.

Taken together, these studies on chromatin factors interacting with, modified by, or phenotypically rescuing TDP-43, indicate a potentially important role of TDP-43 as an epigenetic regulator with a high capacity for modulating chromatin, transcriptional processes, and DNA damage/repair pathways. A synthesis of the identified factors can be found in Table 1.

### 2.2. TDP-43 and Local/Specific Gene Transcriptional Regulation

**Figure 1:**
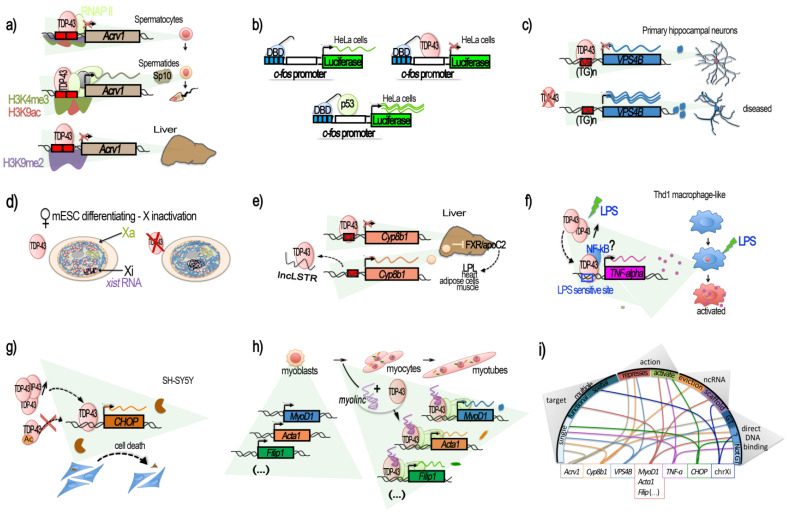
TDP-43-mediated transcriptional regulation. Genes for which TDP-43 has been shown to regulate the transcription by acting at the promoter level are illustrated in their context. (**a**–**e**) Transcriptional repression, involving the direct binding of TDP-43 to the target DNA regulatory region. (**a**) TDP-43 binding on *Acrv1* promoter via two GTGTGT-motifs controls the production of Sp-10 protein during mouse spermatogenesis. TDP-43 at *Acrv1* promoter is still observed when histones acquire activating modifications (H3K9Ac, H3K4me3, increases in RNA-pol II) and transcription starts in spermatids. In the liver, TDP-43 binding and inactive chromatin mark H3K9me2 associates with *Acrv1* inhibition (adapted from [40]). (**b**) Repressive potential of TDP-43 on the *c-fos* promoter. Tethering of TDP-43 to a reporter plasmid using Gal4 DNA Binding Domain (DBD), fused to TDP-43 at Gal4 binding sequences (blue boxes), upstream of the *c-fos* promoter, represses the promoter-induced luciferase expression (adapted from [40]). (**c**) In neurons, TDP-43 represses the promoter of VSP4B, ensuring recycling endosome transport. The repression occurs via the binding of TDP-43 at a GT-rich region less than 1 kb before *VPS4B* TSS. Loss of TDP-43 derepresses the *VPS4B* promoter, leading to loss of dendrites and dendritic spines (adapted from [41]). (**d**) TDP-43 contributes to the supplementary X inactivation (Xi) and X-linked genes in females. TDP-43 interacts with Xist RNA in female cells together with other Xist RNA binding proteins: PTBP1, MATR3, or CELF1. The TDP-43 strongest binding within Xist occurs at the 3′ end of the E-repeat containing multiple (GU)n tracts and persists after completion of X inactivation. Depletion of TDP-43 induces significant nuclear dispersal of Xist and defects in DNA compaction (adapted from [44]). (**e**) TDP-43 binds to a short 40 bp region located from −200 to −160 of *Cyp8b1* promoter in liver and represses its expression. The decrease in Cyp8b1 results in the activation of FXR and an increase in apoC2 levels and diffusion, resulting in enhanced triglyceride (TG) clearance in several mice tissues (muscle, heart, and adipose cells). *lncLSTR*, a liver-specific lncRNA, binds TDP-43 protein and impedes its binding onto *Cyp8b1* promoter, consequently counteracting TG clearance (adapted from [45]). (**f**–**h**) Transcriptional activation. (**f**) TDP-43 binds to and activates the *TNF-alpha* promoter at an LPS-sensitive binding site, located −550 to −487, and mediates the activation of Thd1 macrophage-like. siRNA against TDP-43 reduces the LPS induction of *TNF-alpha* by 50% (adapted from [46]). (**g**) TDP-43 is a direct transcriptional activator of the *CHOP*/*GADD153* promoter in SH-SY5Y, provoking cell death. Binding within the *CHOP* promoter potentially occurs in a region comprised within the bp −300 and −30 from the TSS. TDP-43 also increases *CHOP* mRNA stability. Acetylation of TDP-43 at lysine 145 and 192 impedes TDP-43 activation of the *CHOP* promoter (adapted from [48]). (**h**) During C2C12 differentiation, TDP-43 is tethered by the muscle-enriched lncRNA *Myolinc* to the promoter of several genes linked to the differentiation of myoblasts into myocytes, such as *Acta1*, *MyoD1*, *Filip1*, and others (adapted from [42]). (**i**) Circplot-like summary of the different modalities by which TDP-43 regulates gene expression. TDP-43 can act at “single” or “multiple” targets “functionally” (e.g., the myogenesis pathway) or “spatially” (chromosome X) related. It can be “repressive” or “activating”, involving lncRNAs acting either by “evicting” TDP-43 or tethering it, thus acting as a “scaffold”. Generally, direct binding of TDP-43 on its target’s promoter has been demonstrated. The dependence for DNA binding on GT-rich sequences (“GT-rich”) or not (“Not GT”), when known, is shown, but is has not always been specified (“?”).

Since 1995, when TARDBP was first identified as being able to bind TAR motif within HIV proviral DNA [15], a dozen additional studies identified TDP-43 as a potentially important player in the regulation of other specific genes, according to several modalities (Figure 1).

For example, studies on the testis-specific mouse *Acrv1* gene coding for the sperm acrosomal protein SP-10 led to the discovery that TDP-43 can bind and repress this gene [38,39,40]. At the mechanistic level, TDP-43 binding at the mouse endogenous *Acrv1* was found to occur in vitro via two GTGTGT motifs located within the *Acrv1* promoter and the N-terminal RRM1 domain of TDP-43. TDP-43 was able to tether *Acrv1* at the nuclear matrix, impeding promoter–enhancer interaction, thus acting as an insulator [39] (Figure 1a). Consistently, mutants either lacking or with mutated (F147L/F149L) RRM1 motif failed to repress transcription [40]. Moreover, TDP-43 was found to bind the *Acrv1* gene promoter in several non-neuronal tissues and cell lines, with different intensities, but it was not always able to maintain transcriptional silencing. However, TDP-43 presence in spermatocytes was necessary in order to *Acrv1* silencing at this stage. As the authors mentioned, this suggests that biological conditions exist under which TDP-43 does not act as a transcriptional repressor [40]. The repressor function of TDP-43 was not compromised by HDAC inhibitors in vitro, suggesting that it does not mediate repression by recruiting histone deacetylases [40]; instead, in round spermatids, where TDP-43 is stably bound, the derepression was accompanied by increased levels H3 K4 trimethylation (H3K4Me3) and K9 acetylation (H3K9Ac) with respect to spermatocytes, as well as by the transition from paused RNA Pol II to productive elongation. In the liver, the *Acrv1* promoter TDP-43-mediated repression was specifically associated with histone H3 dimethylated K9 (H3K9me2) [40].

This study also illustrated a potential generic repressor function of TDP-43. This property was demonstrated by artificially bringing TDP-43 in proximity of the *c-fos* core promoter by using a fusion construct between the DNA-binding domain (DBD) of Gal4 protein and TDP-43, and by coding Gal4-binding sequences upstream of the *c-fos* promoter. In HeLa cells, the use of this system led to the repression of the downstream luciferase reporter gene, respective to the use of Gal4 DBD protein alone. Similar constructs with Gal4 DBD fused to p53 protein, on the contrary, enhanced luciferase expression [40], therefore demonstrating an effect specific to TDP-43 (Figure 1b).

Particularly relevant for ALS is the work of Schwenk et al., that revealed a mechanistic link between nuclear loss of TDP-43, TDP-43 gene expression regulatory functions and trophic signaling alteration in neurons [41]. The authors reported that TDP-43 knockdown in neurons from rat and human iPSCs triggered the upregulation of VPS4B mRNA and protein levels up to threefold. In turn, the upregulation of VPS4B inhibited the transport of recycling endosome, impairing the correct surface expression of key receptors for dendrite growth (such as ErbB4, FGFR1, EphB2) and axonal guidance factors (e.g., Robo1, Unc5c/d, EphB2, TrkB). This, consequently, led to loss of dendrites and dendritic spines, potentially compromising synaptic transmission, as observed in ALS. At the mechanistic level, TDP-43 acted as a transcriptional repressor of VPS4B by binding its promoter through a classical TG-rich motif. This effect was demonstrated in vivo by ChIP experiments in rat primary neurons and human brain, and in vitro by luciferase gene reporter assay [41] (Figure 1c).

Another additional mechanism potentially linking TDP-43 nuclear functions to neuronal signaling was recently identified [43]. In this work, the authors identified a new long non-coding RNA (lncRNA) called *neuroLNC* and found it to strictly localize in the cell nucleus and to be implicated in synaptic vesicle (SV) release. *neuroLNC* lncRNA is conserved from rodents to humans, and its expression appears highly restricted to the brain, and more specifically to neuronal cells. Interestingly, mass spectrometry (MS) analysis for protein interactors highlighted TDP-43 as the highest and only highly significant enriched protein interacting with *neuroLNC*. Further IP assays against TDP-43 confirmed their interaction and featured the importance of the *neuroLNC* RNA UG-repeats in the interaction, since a *neuroLNC* with mutated UG-repeats loses its ability to bind to TDP-43. At the functional level, downregulation of TDP-43 abolished the effects of *neuroLNC* overexpression on synaptic vesicles, and the UG-repeats-mutated *neuroLNC* was unable to potentiate SV release. Like TDP-43, *neuroLNC* is chromatin-associated, as shown by DNA ChIRP-seq analysis, and localized chiefly at intronic regions (82%) and at the upstream regulatory regions (13%) of genes. The ChiRP also revealed the binding of *neuroLNC* to several classes of RNA. Gene ontology (GO) analysis of the DNA- and RNA-bound elements highlighted several neuronal genes implicated in neurotransmitter release, synapse organization, glutamatergic signaling, and regulation of neuritogenesis [43]. However, the site of interaction with TDP-43 and *neuroLNC* in the nucleoplasm or on chromatin has not been clearly established, and whether *neuroLNC* promotes the transcription of these genes and/or the stabilization of the mRNAs that are bound will deserve future studies [43]. Finally, it is also notable that pools of RNA and of DNA associating with *neuroLNC* in ChIRP experiments are only partially overlapping [43], suggesting the possibility of several distinct nuclear functions for *neuroLNC* that may not all be related to TDP-43. 

At present, *neuroLnC* is the best-characterized example of a neuron-specific lncRNA- TDP-43 interaction with implications on the regulation of gene/chromatin. However, there are additional examples already documented in other tissues that suggest a wider role for TDP-43–lncRNA interaction. For example, a previous study showed that TDP-43 is part of a proteins–lncRNA Xist condensate, and is required for anchoring Xist to the inactive X (Xi) and for the silencing of the Xi-territory in ESCs [44] (Figure 1d). In addition, both in vitro and in vivo studies performed in mouse liver showed that TDP-43 could directly bind to a short 40 bp sequence in the proximal promoter region (−200 to −160) of the cytochrome P450 8b1 (Cyp8b1) coding gene, a protein regulating triglyceride clearance, and inhibit *Cyp8b1* transcription [45]. Notably, TDP-43 binding was negatively regulated by *lncLSTR*, a liver-enriched nuclear lncRNA with lipid-lowering effects. Direct interaction of *lncLSTR* with TDP-43 was demonstrated via reciprocal pulldown experiments in liver tissue [45] (Figure 1e).

Surprisingly, other examples of recent evidence indicate a role for TDP-43 in the positive regulation of the transcription of other genes. One example is represented by TNF-alpha activation in human monocytic cells THP-1, differentiated into macrophages by PMA and stimulated by LPS [46]. Analysis of the cDNA libraries obtained before and after LPS stimulation by a yeast one-hybrid system and subsequent EMSA did indeed identify TDP-43 as a factor activated by LPS and able to activate *TNF-alpha* transcription by binding an LPS-responsive element within the *TNF-alpha* promoter region (−550 to −487). In this way, TDP-43 was found to act as a mediator of LPS promotion of the pro-inflammatory factor TNF-alpha, a result confirmed by both siRNA knockdown and the overexpression of TDP-43 [46] (Figure 1f). Interestingly, in the experimental setting, the addition of LPS produced a transitory, early-response transcriptional activation of TDP-43 (with mRNA levels peaking at 20 min post-LPS stimulation) that preceded a prolonged TDP-43 protein increase and *TNF-alpha* mRNA upregulation [46]. The authors also showed that NF-kB indirectly binds the *TNF-alpha* promoter, and suggested that TDP-43 could be the factor by which NF-kB reaches the *TNF-alpha* promoter [46]. Indeed, TDP-43 was previously shown to interact with the NF-kB p65 subunit and to act as a co-activator of NF-kB at the NF-kB recognition sequence without direct binding to it [80]. As underlined by the authors, these findings could have implications for TDP-43-linked neurodegenerative diseases as glial cells expressing higher levels of TDP-43 produced more pro-inflammatory cytokines and neurotoxic mediators after stimulation with LPS or reactive oxygen species (ROS) [80]. It is notable that the increase in TDP-43 alone did not trigger inflammation but instead enhanced a hyperactive inflammation response [80].

Another recent study reported a case of TDP-43 behaving as a transcriptional activator using both ChIP and a luciferase reporter assay in SH-SY5Y, this time activating the C/EBP-homologous protein (CHOP) promoter, also known as DNA-damage-inducible transcript 3 (GADD153) [48]. Indeed, it was previously shown that CHOP participates in the cell-death induced by TDP-43 overexpression since the upregulation of TDP-43 overexpression was able to increase the amount of CHOP proteins, both upregulating the *CHOP* mRNA level and attenuating CHOP protein degradation [47]. Recent experiments performed in SH-SY5Y cells by Sanna et al. indicated a direct interaction between TDP-43 and the *CHOP* proximal promoter [48] (Figure 1g). Moreover, activation of the *CHOP* promoter via TDP-43 binding appeared to be negatively modulated by acetylation. Indeed, acetylation-mimic point mutations (KK-QQ), not acetylation-null (KK-AA) in the RRM1–RRM2 region of TDP-43, were found to abolish *CHOP* transcriptional activation. On the contrary, *CHOP* promoter activity was enhanced by HDAC1, which deacetylated WT TDP-43 [48]. Interestingly, in ALS post-mortem tissue, HDAC1 levels have been found to be impaired [81]. HDAC1 and HDAC6 constitute the two HDACs found to modulate TDP-43 toxicity, as mentioned in the first part of this review, thus giving a potential indication that impaired HDAC1 in ALS could disrupt the TDP-43–CHOP cell death induction axis. In any case, this case provides an additional example that TDP-43 can act not only as a repressor or stabilizer of transcription, as has been reported so far. Depending on features yet to be better understood or on local contexts, TDP-43 can directly activate gene transcription. The exact sequences targeted by TDP-43 within *TNF-alpha* and *CHOP* promoters are still unknown.

As shown for TDP-43-mediated gene repression, gene activation can also be mediated by lncRNA-TDP-43 interaction. Thus, in mouse skeletal muscle, the interaction of TDP-43 with a muscle-enriched lncRNA called *Myolinc* appears essential for the binding of TDP-43 to the promoter regions of about a thousand of genes, including essential muscle genes (e.g., *Acta1*, *MyoD1*, *Ccnd1*, *Tnnc1*, *Tnni1*, or *Filip1*) [42] (Figure 1h). Both *Myolinc* and TDP-43 are critical to activating myogenic regulatory networks for the differentiation of myoblasts into myocytes and for the subsequent formation of multinucleated myotubes. Lack of *Myolinc* relocalized TDP-43 to other regions and abrogated activation of the myogenic regulatory network [42]. It is notable that the expression of *Myolinc* has been observed in other tissues, including in the brain, albeit at lower levels. In addition, an siRNA against TDP-43 not only significantly reduced the gene expression levels of these muscle genes but also that of *Myolinc,* suggesting that the *Myolinc* gene itself is also under the control of the TDP-43 protein [42]. Finally, H19 knockdown significantly decreased the enrichment of TDP-43 to the promoter of *MyoD1* in Porcine muscle satellite progenitor cells, thus representing another lncRNA involved in TDP-43-mediated muscle differentiation. Although the precise underlying mechanisms at play are yet to be elucidated, H19 has already been reported to directly interact with TDP-43 [49].

Although the exact mechanisms by which TDP-43 exerts repressive vs. activating gene expression modes are not clear yet, these studies collectively support a limited but potentially important role for TDP-43 as a transcriptional regulator (features recapitulated in Figure 1i), the alteration of which, in addition to that of RNA processing, can severely affect the physiology of the cells.

### 2.3. TDP-43 and Genome-Wide Transcriptional Regulation

Cellular evidence for a potential broad function of TDP-43 in transcriptional regulation in physiological conditions has been supported by confocal and electron microscopy studies combined with in situ detection of transcription [82]. In this work, TDP-43 was found distributed throughout the euchromatin of the primary sensory ganglia neurons of rats and to be enriched at perichromatin fibrils, i.e., mRNA transcription and processing sites. In particular, TDP-43 signal was evident at sites of nascent pre-mRNA. Conversely, only weak TDP-43 immunolabeling was found in nuclear speckles that represent areas enriched by splicing factors. Finally, transcriptionally silent constitutive centromeric and telomeric heterochromatin, as well as Cajal bodies, did not concentrate TDP-43 [82]. These microscopic observations globally concur with chromatin fractionation analyses followed by Western blot experiments performed in HeLa cells [24].

About 7 years later, a higher resolution of the genome localization of TDP-43 was first given on its Drosophila homolog, TBPH, by a plethora of experiments based on ChIP-seq, RNAi depletion, transcription blockade, affinity chromatography, and immunoprecipitation conducted in Drosophila [83]. This study confirmed the presence of TDP-43/TPBH at gene regulatory locations, where TBPH appeared to bind chromosomes at specific sites, and not only at splicing-related features, such as gene bodies, but also at genes, enhancers, and Polycomb response elements (PREs) bound by cohesin. At these regulatory regions and genes, TBPH was found to ensure high levels of Nipped-B and cohesin on the sites [83]. As described in mouse or human, TBPH targeting has been linked to the presence of TG reach repeats in the non-template strand of these genes. Based on the obtained results, a model was proposed forecasting that UG repeats on the nascent transcripts recruit TDPH via RRM1 domain binding, then the Nipped-B and cohesin complex are recruited. In turn, Nipped-B boosts the TDPH presence at poorly-transcribed regulatory regions, such as enhancers and PREs. Continued transcription is not required to maintain their binding once it has been established [83]. In higher organisms, such as human and mouse, PRE are not conserved, and the mechanisms and modalities of Polycomb-repressive complexes, PRC2 or PRC1, loading to chromatin are various and multifactorial and still represent a field of intense research [84,85,86,87]. The colocalization or interaction between TDP-43 and PRC2 or PRC1 have not been reported yet, but PRC2 target genes were recently found derepressed in post-mortem brain samples from ALS/FTD patients with *C9orf72* (C9) repeat expansions, and the PCR2 HMT subunit EZH2 was found largely insoluble [88]. Interestingly, a deep characterization of the extent of TDP-43 associations with chromatin in the human was recently obtained via the analysis of ENCODE-released ChIP data in HEK293T cells [89]. TDP-43 general genome-wide localization at gene promoters was confirmed. In particular, a strong enrichment was observed at promoters in association with high RNA Pol II presence. However, TDP-43 did not bind, at least directly, RNA Pol II, nor did it string along with it within the gene body of active genes [89]. siRNA-induced silencing of TDP-43 reduced the transcription of thousands of genes, as analyzed by GRO-seq, including protein-coding, antisense non-coding, and lincRNA genes, and conversely, it activated only a little fraction of genes in each of these categories. Several miRNA and snRNA were also affected [89]. Still, there was no compelling evidence of a relationship between TDP-43 abundance at a gene promoter and the degree of transcriptional change after TDP-43 loss. Instead, TDP-43 loss resulted in increased transcription of repetitive elements found within expressed genes belonging to the Alu class of non-autonomous retrotransposons, the affected density of which corresponded to changes in gene transcription [89]. As TDP-43 did not appear to interact with RNA Pol II, nor with the affected Alu at these regions, the mechanisms behind it may be indirect, affecting other TDP-43 pathways.

At the beginning of 2021, Maor-norf and colleagues, working on mouse cortical culture and combining ATAC-seq and RNA-seq, investigated the consequences on the chromatin accessibility of ALS-related protein overexpression [90]. Though the main outcome of the publication relies on the *C9orf72* poly(PR) ((PR)_50_) mutant and the interesting finding that (PR)_50_-induced neuronal death can be dampened via p53 inhibition, the authors nonetheless obtained very interesting findings regarding TDP-43 neuronal overexpression. Despite the neurodegeneration looking “grossly similar”, the modifications of the underlying chromatin and gene expression programs were different. TDP-43 and *C9orf72* (PR)_50_ conveyed unique chromatin and transcriptional footprints. Loss of chromatin accessibility was observed for all lentiviral-treated cultures, even the GFP control; however, a gain in chromatin accessibility was observed for TDP-43 after 60 h and associated with a chromatin more accessible for a variety of TFs, as evidenced via ChromVar, and several gene co-expression networks were deregulated [90], findings that definitively deserve deeper characterization.

As a result of these investigations, it appears that TDP-43 has a broad ability to affect the transcription of all categories of genes, from coding to non-coding classes, to a degree that is greater than had been previously appreciated. This can be achieved indirectly through the modulation of genes involved in chromatin remodeling and transcription, or directly via association with genes promoters. While direct, multimodal regulation, either repressive or activating, has, as discussed above, been reported for several genes, understanding of the general function of TDP-43 at the TSS of active genes genome-wide still requires further investigation. It was proposed that the transcription-independent binding of TBPH, and hence possibly of TDP-43, could serve to reduce the fluctuations in the levels of transcription over time, with the intriguing possibility that the aggregate-prone low-complexity C terminal domains in TBPH might also facilitate enhancer–promoter looping or loops stabilization [83].

Remarkably, Nie and colleagues reported very recently on the requirement of the maternal (oocyte) TDP-43 protein to activate the zygotic genome during embryogenesis by promoting RNA Pol II transition from a paused to an elongating state [91]. Experiments driven in mouse showed that maternal TDP-43 proteins translocate from the cytoplasm to the nuclear space from the 2C stage, where they localize at RNA Pol II clusters and associated with RNA Pol II, as shown via a proximity ligation assay (PLA), co-IPs and ChIP-seq-like Stacc-seq technology. TDP-43 also co-occupies with RNA Pol II at the promoters of ZGA genes at the late 2C stage. Importantly, the deletion of maternal TDP-43 led to defective zygote genome activation. Indeed, their results support the fact that TDP-43 promotes the expression of ZGA genes by activating transcription of RNA Pol II elongation from its pausing through RNA Pol II CTD Ser2 Cyclin T1 phosphatase during mouse maternal-to-zygotic transition [91]. However, as observed for *Acrv1* gene promoter activity during mouse spermatogenesis [40], the here-described essential role of TDP-43 in early mouse embryogenesis is stage-specific, as its absence at an earlier stage, i.e., in mouse full-grown oocytes, only mildly affects gene expression [91]. Overall, TDP-43 seems to have a pivotal role in the cell fate gene induction of different tissues (spermatogenesis [40], myogenesis [42], and embryogenesis [91]).

### 2.4. TDP-43 and DNA Repair

Alongside the TDP-43 function in gene transcriptional regulation, several recent studies have strengthened the potential role of TDP-43 in genome stability and DNA repair [23,92,93,94,95,96]. 

Upon DNA damage induced in pluripotent stem cell (iPSCs)-derived motor neurons from a healthy subject, or in differentiated neuronal line SH-SY5Y, normal TDP-43 is rapidly recruited at double-stranded breaks (DSB) sites. TDP-43 was shown to stably interact with DNA damage response (DDR) and neighbor homologous end-joining (NHEJ) repair factors. Specifically, it acted as a scaffold protein for the DDR complex (γH2AX, pATM, Ku70, p53BP1) and the break-sealing XRCC4-DNA ligase 4 complex (XRCC4, lig4, XLF), mediating its recruitment at induced DSB sites [23,93]. 

In vitro experiments further showed that TDP-43 can directly bind dsDNA oligonucleotides with free blunted ends, but not if ends are biotin-blocked or in case of a ssDNA break [23], thus supporting the TDP-43 recognition and binding of DSBs in the genome. It is notable that TDP-43 was found pre-complexed with some proteins of the repair machinery, i.e., Ku-70, Ligase 4, and XRCC4, but also with p-53BP1 and γH2AX, already in the absence of artificially induced breaks [23]. Upon DSB induction, these interactions were significantly enhanced [23]. The authors showed that TDP-43 specifically interacted with NHEJ proteins and remained at DSB until repair completion [23]. In SH-SY5Y, TDP-43 Q331K mutant prevented the nuclear translocation of XRCC4-DNA ligase 4, and cells showed elevated levels of reactive oxygen species, thus contributing to both DNA damage production and irresolution [93].

**Figure 2:**
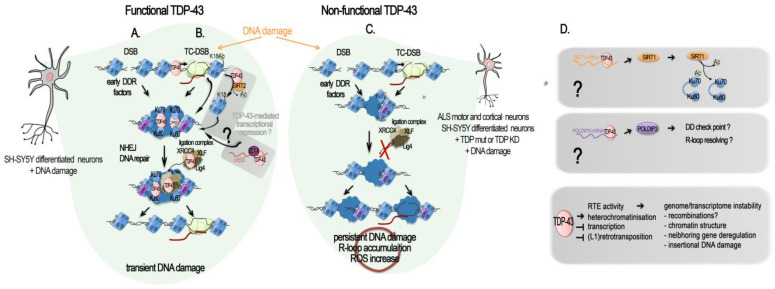
TDP-43-mediated DNA repair: direct and indirect roles. Schematic diagrams of TPD-43 role in DSB repair. (**A**–**C**) Direct role of TDP-43 in DSB repair and its misregulation in ALS. (**A**) TDP-43 in DNA double-stranded break (DSB) repair. TDP-43 interaction with activated (phosphorylated) DNA damage repair (DDR) response factors (p-ATM, p-53BP1, p-H2AX = yH2AX) facilitates the NHEJ repair in neurons by supporting the recruitment and activity of the XLF/XRCC4/Lig4 complex [23]. (**B**) TDP-43 in transcription-coupled DSB (TC-DSB) repair. TDP-43 interacts with several key factors in the transcription-coupled repair (i.e., DHX9, COPS3/4, AQR, RFC, PARP1, XRCC1, TDP1, APEX1, Ku70/80, and condensin SMC3), and binds non-blocked dsDNA ends such as those created at DSB. It is also probably involved in the transcriptional silencing following DSB through its recruitment of SIRT-2 and subsequent H3K18 deacetylation, as evidenced in HeLa and MEFs cells [97] and in post-mitotic neuronal cells. The hypothetical recruitment of RNA helicase DDX5 with TDP-43 at R-loop by the *Lnc530* to resolve their aberrant formation is still to be investigated in neurons and in the human. (**C**) TDP-43-related genome damage in ALS motor and cortical neurons and in differentiated neuronal SH-SY5Y cells. In presence of a mutant or mislocalized TDP-43, yH2AX levels are reduced, and the NHEJ complex (XLF/XRCC4/Lig4) is not recruited to damage sites for repair, resulting in an accumulation of damaged DNA and leading to neurodegeneration. (**D**) TDP-43 regulation of genes impacting DNA damage and repair. TDP-43 positively regulates *Sirt1* and *Poldip3* mRNA levels by binding to their 3′UTR, stabilizing them. Upon TPD-43 alterations, *Sirt1* mRNA levels decrease and SIRT1-mediated deacetylation of Ku70 is reduced, lowering HR and NEHJ DSB repair. Likewise, a decrease in *Poldip3* mRNA levels could reduce the DNA damage checkpoint and the resolution of R-loop. SIRT1 and POLDIP3 functions in the DSB response in mature neurons remain evasive. Different means by which functional TDP-43 acts against RTE activity at the chromatin level are listed, as well as the general negative consequences which RTE uncontrolled activity can have on the genome and transcriptome stability.

As expected from these findings, deprivation of TDP-43 led to an accumulation of DNA damage. As such, TDP-43 deprivation in cycling iPSCs-derived NPCs and SH-SY5Y conveyed an accumulation of endogenous DSBs, despite DDR activation, with an increase in γH2AX, p53BP1, and pATM at 96 h after TDP-43 KD [23]. Subsequently, cells proceeded to apoptosis [23]. This TDP-43 function is universal among metazoans as TDP-1-lacking worms also have an impaired DSB repair [23]. An increased in yH2AX upon TDP-43 shRNA was also observed in differentiated and not differentiated SH-SY5Y, as well as in NPC-derived motor neurons [23], and in SH-SY5Y cells overexpressing a A382T TDP-43 mutant [97]. However, TDP-43 knockdown from NSC-34 motor neuron-like cells or primary cortical neurons resulted in a significant decrease in both γH2AX foci and global γH2AX amounts [95], thus underlying probable cell-type and assays specificities. Since neurons are post-mitotic cells, they are particularly dependent on the NHEJ DNA repair pathway, unlike other cells that can take support from the less-error-prone DNA repair by homologous recombination (HR) [98]. Two mechanistically distinct NHEJ DNA repair pathways exist: the classical (C-NHEJ) is Ku70, Lig4, and Rad51-dependent [99]; the alternative is NHEJ (alt-EJ), which is not dependent on these factors [99]. From the evidence presented above, and further sustained by an additional study using GFP-reporter systems specific to either of the two NHEJ DSB repair mechanisms, TPD-43 clearly participates in classical NHEJ DSB repair [95]. On the contrary, neither mutant (Q331K, A315T) nor wild-type TDP-43 participate in the modulation of the alt-EJ [95]. TDP-43’s role at DSB sites for NHEJ repair and related dysfunctions in contexts of altered TDP-43, such as ALS, is illustrated in Figure 2A–C. 

Additional insights into the protective role of TDP-43 against DNA damage and the mechanism behind it come from a recent work on the bacterial pathogen *Listeria monocytogenes* [96]. Upon infection, *Listeria monocytogenes* causes SIRT2 accumulation in the nuclear and chromatin spaces. SIRT2 is a deacetylase and its translocation to the chromatin provoked a global loss of H3 Lysine 18 acetylation (H3K18ac), a mark enriched at the TSS of transcriptionally active and poised genes. On a local scale, both SIRT2 and H3K18ac were redistributed. SIRT2 enriched at the TSS of a large subgroup of genes that lost H3K18ac and became repressed, and get depleted at other genes that gained increased H3K18ac and became activated [100]. Eldridge and Hamon found that 72% of the genes that gained SIRT2 and become repressed upon infection have TDP-43 at their TSS and showed that TDP-43 interaction with SIRT2 is essential for its enrichment at the TSS and H3K18 deacetylation during infection [96]. Mechanistically, SIRT2 and TDP-43 interact in the basal state of the cells. However, upon infection, interaction between SIRT2 and TDP-43 increases, partially due to SIRT2 phosphorylation, and SIRT2–TDP-43 complexes are loaded onto their targets TSS, with TDP-43 serving as a scaffold for SIRT2 [96]. As observed in case of induced DNA damaged in motor neurons, TDP-43-targeting genomic DNA was dependent on the presence of DNA:RNA hybrids called R-loops [92]. In the absence of TDP-43 or SIRT2, SIRT2-mediated H3K18 deacetylation did not occur and host DNA damage caused by infection accumulated, thus showing a protective role for TDP-43 against DNA damage [96]. In the brain, contradictory roles of SIRT2 as both neuroprotective and neurotoxic have been reported [101], but its implication in DNA damage and TDP-43-mediated DNA repair have not yet been investigated (Figure 2B).

In addition to a direct intervention of TDP-43 at DSB site, TDP-43 was shown to be important for the production of two proteins involved in DNA repair, SIRT1 and POLDIP3 (DNA Polymerase Delta 3, Accessory Subunit). SIRT1 is a Sirtuin implicated in dsDBR and is required for cell survival. RNA-IP and RNA pull-down assays in human neuroblastoma SH-SY5Y and embryonic kidney HEK293T cells demonstrated that TDP-43, in complex with FMRP (fragile X mental retardation protein) and STAU1 (Staufen) proteins, specifically binds to the 3′-UTR of SIRT1 mRNA and positively regulates its stability and hence its protein production [102] (Figure 2D). In a cellular model myeloid leukemia K562, inhibition of SIRT1 impeded Ku70 deacetylation and consequently impaired NHEJ DDR [103]. Despite the demonstration being conducted in cycling cells, SIRT1 implication in the NHEJ DDR pathways could also be effective in cells post-mitotically, and it could be linked to its protective roles in several neurodegenerative diseases, including Alzheimer’s, Parkinson’s, and ALS [104]. On the other hand, POLDIP3 plays critical roles in disassembling R-loops genome-wide and activating the DNA damage checkpoint [105], and its transcript is one of the well-characterized TDP-43 targets. In particular, inclusion of *POLDIP3* exon 3 was significantly altered in different cell lines depleted for TDP-43 and other hnRNPs linked to TDP-43 functions [59,77,78], as well as in various motor regions of CNS of ALS patients [106]. Although the significance of this variant has not been elucidated in detail, different studies suggest its role in cell size [59,106]. However, implications in DNA repair and DNA damage checkpoint may not be excluded due to the multitude of POLDIP3 functions across the RNA and DNA metabolism (Figure 2D).

Interestingly, a role in the prevention and/or repair of DNA damage has also been proposed for FUS, another well-characterized fALS-linked protein, both in the motor neuron-differentiated neuronal cell line and in non-neuronal dividing cells [92,94,107,108]. In dividing cells, TDP-43, FUS, and the DNA damage-repair protein, BRCA1, localize together at sites of active RNA polymerase II transcription-associated DNA damage. The depletion of either was shown to trigger an increased sensitivity to transcription stalling agents and DNA damage [92,94]. Interactome analysis of FUS and TDP-43 by affinity enrichment mass spectrometry in HeLa Kyoto cells further revealed binding to several factors important to DNA repair mechanisms that can be replication-dependent, -independent, or both, common to FUS and TDP-43. These included chromatin-associated proteins and transcription-coupled DNA repair proteins, as well as nuclear RNA exosome and ribosome. While interaction levels of these factors with TDP-43 were stable before and after treatment with the DNA damaging agent etoposide, the interaction of FUS with TDP-43 and these factors increased. DNA damage also triggered an increase in G-protein-coupled receptor interaction with TDP-43 [94]. Notably, TDP-43 appeared to be more essential to genomic stability and DNA damage repair than FUS [94].

Apart from their gene regulatory function, R-loops can also function to promote DNA repair, particularly in the context of transcriptionally coupled repair [109,110]. Interestingly, in silico analysis shows that many SIRT2-regulated sequences contain or are predicted to contain R-loops [100]. Additionally, there are multiple studies demonstrating that TDP-43 localizes to and interacts with R-loops (see DNA repair section and [97,111]). One of them further sustains the role of TDP-43 in genome integrity, showing that TDP-43 prevented genome-destabilizing R loop-accumulation in neuronal and non-neuronal cells, and in patients cell lines [97]. Mislocalization of mutated TDP-43 (A382T or G294V) caused R-loop accumulation, R-loop-dependent increased DSBs, and Fanconi Anemia repair centers [97]. Thus, TDP-43 depletion not only caused R-loop-accumulation and R-loop-dependent DNA damage but resulted in the accumulation of the transcription-replication collision-associated FANCD2 repair foci [97]. In agreement with these findings, analysis of ChIP-seq and RNA-seq data from K562 erythroblastoma cells confirmed the co-localization of TDP-43 at expressed genes and, in particular, at R-loop-prone expressed genes, while only a small proportion of silent genes held TDP-43 [96,97]. In all cases, TDP-43 predominantly localized at the TSS. Over-expression of the wild-type form of TDP-43 in human SH-SY5Y cells caused local but not genome-wide R-loop accumulation and no significant increase in γH2AX foci, in accordance with a sensible nuclear loss of the endogenous TDP-43 [97]. 

The key role of TDP-43 in preventing R-loop accumulation has been further highlighted in the recent work of Gong et al. [112]. Studying the control of R-loop formations in mouse embryonic stem cells (mESC), they found that a long non-coding RNA, namely, *Lnc530,* localizes to R-loops, controls their levels, and preserves genomic stability. To understand how *Lnc530* regulates R-loops, they performed in vivo RNA pull-down with MS analysis and found two strong candidates, DEAD-box RNA helicase 5 (DDX5) and TDP-43, with whom the *Lnc530* forms a DDX5- *Lnc530*-TDP-43 complex that prevents unwanted R-loop formation and elevates the concentration of DDX5 and TDP-43. [112]. RNA-pull-down and reciprocal co-IPs with KD of either of the three components demonstrated the inter-dependent formation of the DDX5-*Lnc530*-TDP-43 complex, probably elevating the local concentrations of DDX5 and TDP-43 to regulate the resolving of R-loops [112]. While *Lnc530* expression is much less abundant in differentiated cells, its ectopic expression in such cells effectively increased the recruitment of DDX5-TDP-43 at R-loops and reduced their aberrant formation [112]. Interestingly, the authors reported having detected abundant *Lnc530* expression in different brain regions of mice at even higher levels than that in mESCs. If *Lnc530* participate in TDP-43, R-loop regulation in mice brain is should be further examined. Similarly, the functionality of the human *Lnc530*, reported to show only partial conservation with mice *Lnc530* [112], and it association with TDP-43 in the human, are to be investigated (Figure 2B).

The link between TDP-43 function in DNA stability and ALS features was also supported by the fact that the spinal cord DNA of a ALS patient presenting the TDP-43 Q331K mutation showed a higher level of γH2AX, a DNA single- and double-stranded break marker, compared to age-matched controls [93]. In the SH-SY5Y neuronal cell line, mutant TDP-43^Q331K^ had a reduced interaction with XRCC4 and Ligase 4, both in unstressed and irradiated cells, and prevented XRCC4-Lig4 nuclear translocation. The authors showed that in addition to defective DNA repair, Q331K expression induced ROS stress, at least in cycling cells, thus fueling the vicious cycle [93]. Loss of DNA integrity was observed in the spinal cords from a cohort of 10 sALS patients but not in controls [23]. This was associated with increased γH2AX foci and DSBs compared to controls. In all ALS spinal cord specimens, an extranuclear increase in TDP-43 was observed, in association with an increase in TDP-43 aggregation and in short fragments, as well as a reduced amount of monomeric forms, thus implicating a depletion of TDP-43 from nucleus/chromatin [23]. Finally, a defect in the repair machinery, as demonstrated by the inhibition of the classical NHEJ repair, led to the delocalization of TDP-43 to the cytoplasm, thus emphasizing a crucial crosstalk between TDP-43 and NHEJ repair machinery in neuronal genome stability [95] (Figure 2C).

Finally, in the report from Guerrero and colleagues, it is important to note that the Q331K mutation of TDP-43 was present in ∼10–20% of total genomic DNA isolated from the sALS patient spinal cord. It was also absent in other brain regions such as the occipital lobe. This suggests that the mutation can be acquired sporadically [93] and somatically, a characteristic that may account for the mosaicism of the disease presentation.

### 2.5. TDP-43 and Regulation of the Genome Dark Matter

**Figure 3:**
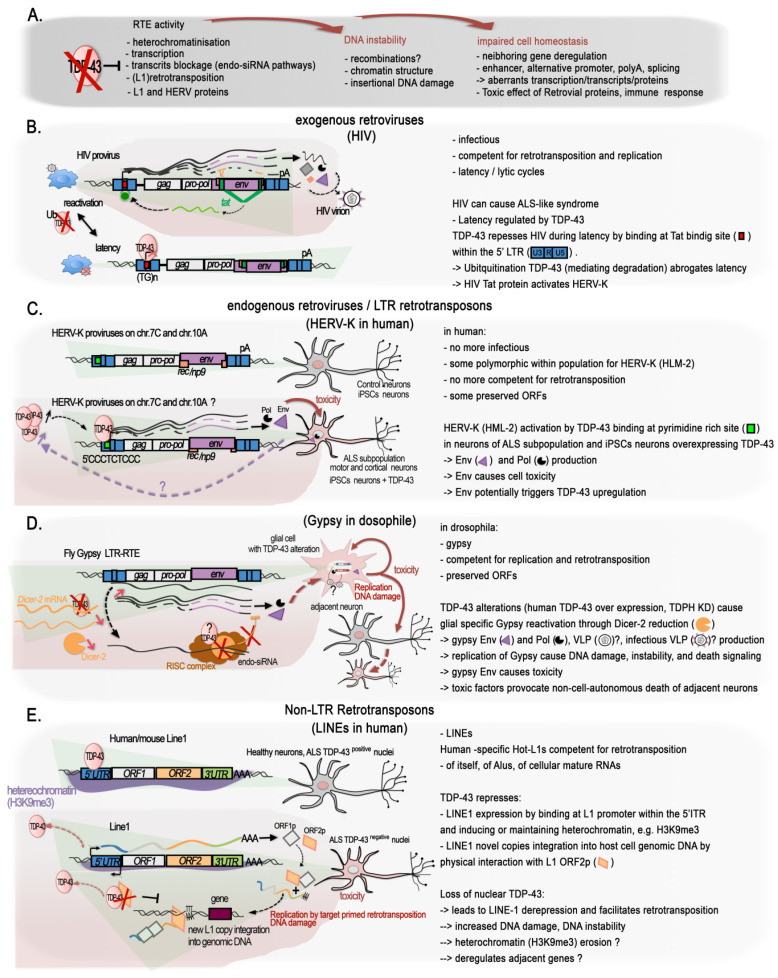
TDP-43 connections to RTE detrimental action in ALS. (**A**) Overview of the general consequences of TDP-43 alterations in DNA stability and cellular homeostasis due to RTE inhibition failure. (**B**–**E**) TDP-43 impact on ALS through the misregulation of RTE. In the cartoons, regulatory sequences (promoter regions), i.e., LTRs in exogenous and endogenous ERVs, and 5′UTR in Lines, are in blue; retroviral gag, pro, and pol genes produced from the same (polycistronic) transcript are in white; and the *env* gene produced by an alternatively spliced transcript is in purple. ORFs for accessory proteins (Tat in HIV, rec and np9 or rec in HERV-K) are depicted. Specificities of each class/specific elements and impact on ALS are listed on the right of each cartoon. (**B**) TDP-43 is able to repress HIV-1 provirus activation. TDP-43 binds to the TAR binding site within the 5′LTR R region and represses transcription. This binding was shown to impede the binding of TAR RNA and Tat-activating protein. A reduction in TDP-43 binding (by ubiquitination and proteosomal degradation) can reverse HIV-1 provirus latency, potentially leading to the production of infectious viral particles (HIV virion). It is known that HIV-1 can promote ALS-like symptoms. HIV can also activate HERV-K elements, notably via Tat [155]. (**C**) TDP-43 overexpression binds to and activates specific HERV-K HML-2 provirus(es), producing the toxic env glycoprotein HERV(HML-2). The HERV-K proviruses found activated in ALS cases (ALS neurons shows immunoreactivity for HERV-K Env) could be HERV-K C7-C and HERV-K C10-A, which are polymorphic proviruses in the human population. TDP-43 binds to the LTR5Hs sequence at a polypyrimidine track in the U3 region (5′-CCCTCTCCC-3′) with high affinity and is able to activate the LTR-promoted transcription. Conversely, HERV-K Env potentially triggers TDP-43 upregulation. (**D**) In Drosophila, the failure of TDP-43 to indirectly repress *Gypsy* retrovirus, a family of endogenous LTR-retrotranposon, leads to cell autonomous and non-autonomous toxicity. The family contains copies with preserved ORFs, capable of retrotransposition and replication. TDP-43 alterations (hTDP-43 overexpression or fly TDP-43 homologue TDPH null) induce the activation, specifically in glial cells, of *Gypsy* copies. It is not clear if replication involves infectious or non-virus-like particles (VLP). Mechanistically, TDP-43 binds to and positively regulates Dicer-2 (Dicer in the human) mRNA and protein. Dicer-2 in the RISC complex controls *Gypsy* and other RTE activity via the endo-siRNA pathway, inhibiting or impeding transduction by promoting mRNA degradation. Lack of TDP-43 by reducing Dicer-2 levels impedes endo-siRNA-mediated control (see text for more detail). (**E**) TDP-43 nuclear loss in the neurons of ALS patients induces L1 expression. TDP-43 represses human L1 by at least two mechanisms: (i) by binding at the 5′UTR promotes and maintaining L1 heterochromatinization; and (ii) by interacting with the ORF2p in cases of L1 retrotransposition, inhibiting the pasting of new copies into the host genome.

The role of TDP-43 in genome stability might not only be linked to the protection of DSB at active transcription, i.e., at transcribed genes; growing evidence shows that it has a function in maintaining silent the so called “Dark matter” “selfish DNA” of our genomes (Figure 2D and Figure 3A). In their recent work on the post-mortem brain of FTD patients, Liu and colleagues integrated their transcriptome analyses with ATAC-seq to examine changes in chromatin accessibility in TDP-43-negative nuclei relative to TDP-positive nuclei from the same samples for seven FTD and FTD–ALS brains [65]. They identified 3457 significantly differentially accessible genomic regions, the great majority (75.2%) of which corresponded to a more closed chromatin in TDP-43-negative nuclei. However more accessible chromatin was enriched for elements typically found in heterochromatic regions, depleted from classical genes, suggesting a similar overall euchromatinization in the TDP-43 pathologic nuclei similar to the one observed in mutant mice [64,65]. The ability of TDP-43 to maintain genome dark matter silence is not so surprising given that it was discovered as a transcriptional repressor of the HIV provirus (Figure 3B).

Mammal genomes are full of remnants from ancient retroviral infections of the germ line cells that have resulted in the integration of proviral genomes into the DNA of offspring. Over time, some of these integrations led to the fixation of the proviruses in the gene pool of the host population, thus becoming an endogenous retrovirus (ERV). In parallel, their subsequent within-germline propagations by means of retrotranspositions or reinfections (copy–paste-like mechanisms) over millions of years led to the formation of several multicopy families that group under the long terminal repeat (LTR) class of TEs. Retroviral ORFs (gag (viral core proteins), pro/int/pol (enzymatic proteins: protease integrase, reverse transcriptase polymerase), env (envelope glycoprotein), and accessory proteins) accumulated disruptive nonsense mutations and proviruses often recombined leading to solo-LTRs. In the human, HERV-derived copies and fragments represent about 8% of our genomic DNA [113,114,115,116]. Insertional and recombinational polymorphism of some HERV copies exists within the population, and they belong to the more recent HERV-K HML-2 family [117,118,119]. Several ORFs from different HERV families still remain, and various examples of domesticated ERV proteins have been reported, especially for env glycoproteins [120,121,122,123]. In addition, LTRs contribute greatly to “cellular” gene regulatory sequences such as promoter, enhancer, or polyadenylation signals [116,124]. In contrast to their human counterparts, some murine endogenous retroviruses (mERV) proviruses still can synthesize infectious particles and retrotranspose.

The long interspersed elements (LINEs) constitute another important class of retrotransposons; they are able to conduct autonomous self-propagation via another copy–paste mechanism thanks to their ORFs encoding ribonucleoproteins, endonuclease, and reverse transcriptase. They account for 17% of the human genome, and several of them, albeit representing a small fraction, are polymorphic within the population [125]. They directly contribute to the expansion of short interspersed elements (SINEs), another class of TE, non-autonomous, and of pseudo- and retrogenes in the genome [126,127]. An additional class of TE, the DNA transposon, mobilizes through a cut–paste mechanism. Together, the TEs occupy nearly 46% of the human genome and 39% of the mouse genome [113,128]. Contrary to human ERV, retrotransposition-competent (RC) copies of LINES in human and mouse genomes are numerous. A small number of these RC-L1s loci, Hot-L1, are highly active [125], notably in human and mouse developing brains [129,130], and result in normal brain genome mosaicism [131].

Overall, TE subfamilies are species specific, but they rely on the same molecular mechanisms for their control and propagation [132], notably epigenetic mechanisms, including CpG DNA methylation. Indeed, genomes evolved defenses against their detrimental potential, and TE are generally silenced by DNA methylation and heterochromatin marks such as H3K9me3, acting as major barrier against their activation [133,134,135,136]. Thanks to the efforts of recent studies, TDP-43 has recently been shown to play a role in regulating them at several levels.

Being highly repetitive by nature, TEs are routinely dismissed from deep-sequencing analyses if not under specific focus, although new sequencing modes have greatly improved their mappability. However, by reanalyzing a series of deep sequencing datasets from RIP-seq and iCLIP-seq from normal brains of rat, mouse, and human, the group of Dubnau uncovered an extensive binding of TDP-43 to TE transcripts [137]. In this way, several ERV/LTR classes, but also SINEs and LINEs and some DNA repeats, were identified. Interestingly, although peaks that map over RefGene (classical “cellular” protein-coding and non-coding genes) annotations were similarly distributed for both FUS and TDP-43 iCLIP-seq experiments in mouse, only TDP-43 clearly targeted TE-transcripts for binding, and via a similar sequence motif (UGUGU), as reported for “cellular” transcripts [137]. 

Importantly, this physiological binding of TDP-43 to TEs was observed to be altered in at least two of the major TDP-43 proteinopathies: FTLD and in a specific subtype of ALS [137,138]. In FLTD patients, reanalysis of iCLIP-seq data showed a reduced association between TDP-43 and TE transcripts for all major classes, including SINE, LINE, LTR, and a few DNA transposon elements, principally originating from intergenic locations [137]. This reduction was more pronounced for TE than for “cellular genes”. Furthermore, TDP-43 depletion and overexpression (acting as a dominant-negative) in mouse brain both conveyed the robust overexpression of some tens to several hundreds of copies of TE-derived transcripts; with the vast majority of them corresponding to those identified in the iCLIP-seq data [137]. With regard to ALS, the dominant feature characterizing a specific sub-group consisting of 20% of ALS patients was a marked retrotransposons re-activation [138]. This recent study applied machine learning-assisted analysis of RNA-seq from frontal and/or motor cortex samples of a cohort of ALS patients and controls [138]. Again, this ALS-TE subgroup included TEs from the LINE, SINE, and LTR classes, as well as several individual retrotransposons from the HERV-H, LINE L1M2a, and L1PA6, and SINE–VNTR–Alu (SVA) families specifically characterized the ALS-TE group. Remarkably, as the authors observed, ALS-TE subgroup was characterized by the lowest TARDBP expression. Additional pathways consistent with TDP-43 functions, such as the depletion of spliceosome and proteosome-linked genes were specifically depleted in the ALS-TE subgroup. Transcriptionally altered epigenetic regulators, namely, chromodomain-helicase-DNA-binding protein 5 (CHD5), lysine acetyltransferase 2A (KAT2A), and the histone H3K4 lysine methyltransferase 2B (KMT2B), were also part of this subgroup [138], in line with the impact of TDP-43 on global histone modifications reported above (see Section 2.1: TDP-43 Is a Global Epigenetic Modifier). The other subgroups displayed either more sustained alterations in the oxidative stress markers, including *SOD1* mRNA (61%), or a strong bias for inflammation and pan-glial cells activation (19%).

These conclusions were broadly supported by analyses performed in human SH-SY5Y neuroblastoma cells, where the use of CLAM, a tool designed to handle repetitive reads on sequencing data of TDP-43-bound RNA obtained by using enhanced cross-linking and immunoprecipitation (eCLIP-seq), unmasked 439 TE-derived RNA bound to TDP-43, corresponding to 31% of all mapping reads [138]. Specifically, 58% of the TE associated peaks (17.6% of the whole TDP-43 bound RNAs) mapped anti-sense with respect to the TEs, as already observed previously specifically for LINE-1 and Alu elements [35,139], and could provide regulatory sequences for the host genes they lie within [138]. In addition, knocking down TDP-43 using an shRNA in SH-SY5Y altered the expression level of several TE, mainly from the LTR class. All the significantly altered retrotransposon transcripts were upregulated, and only a fraction of which was identified by eCLIP-seq under normal TDP-43 expression [138], thus providing further evidences that TDP-43 normally contributes to the silencing of retrotransposon transcripts, and that this can be achieved at the RNA and DNA level.

All these data support a specific functional and conserved role for TDP-43 in the repression/regulation of TE elements. Importantly, misregulated TE expression can have a number of detrimental impacts on chromatin, such as those observed in ALS and other neurodegenerative diseases. They may include genome instability via the spurious integrations of new repeats, activation of the DNA-damage stress response, or deregulation of the neighboring genes (Figure 3A).

#### 2.5.1. The Singular Case of Human HERV-K Env Protein Activated by TDP-43

An increased expression of the primate specific ERV-K family in a subgroup of sALS patients was reported in at least two studies [140,141], and TDP-43 was proposed to behave as an activator rather than a repressor. HERV-K overexpression of selected HERV copies was observed specifically to occur in the cortical and spinal neurons of some of the sALS patients, but not of healthy individuals, AD, or PD patient brains. The first study, looking for *pol* gene containing mRNA, identified several actively transcribed loci in the HERV-K HML-2 and 3 subfamilies, including specific copies with protein coding potential lying within a candidate interval for MND, in which the susceptibility genes were not identified [140]. Expression of the RT protein was observed specifically in ALS brains and localized to cortical and motor neurons [140]. In the second study, starting from an env perspective [141], an increase in ALS patients of env containing transcripts specifically from the family HERV-K was found. Immunostaining confirmed the high expression of HERV-K env protein in the cytoplasm of pyramidal cortical and spinal neurons in these ALS patients, but not in glial cells and not in healthy or AD affected tissues [141]. Further suggesting a possible connection with TDP-43, the ectopically driven TDP-43 expression in human stem cells-derived neurons increased the expression of in all *gag*, *pol*, and *env* retroviral genes regions in a correlated dose-dependent manner, supporting the activation of proviral forms of HERV-K [141], or the activation of multiple copies holding the same 5′LTR regulatory sequences.

Mechanistically, the knockdown of endogenous TDP-43 with siRNA reduced HERV-K expression, thus arguing against a derepression of a HERV-K copy (or copies) caused by overexpression-mediated nuclear depletion of TDP-43 in the neurons of these patients or in vitro. In addition, ChIP assays, together with in vitro luciferase assays on the HERV-K LTR in HeLa cells, confirmed the activating effects of TDP-43 load and binding onto the LTR [141]. Furthermore, TDP-43 binding correlated with association of RNA Pol II p-Ser2, a processive form, on the consensus LTR from the HML-2 LTR5Hs-holding LTR-type subgroup. Interestingly, high affinity binding happens at a non-canonical polypyrimidine track (5′CCCTCTCCC) within the LTR region (+726), and less strongly at four other polypyrimidine motifs along it [141]. It is to be noted that the ChIP assays were performed on a plasmid holding a prototypal HERV-K LTR, and we require a final confirmation that the HERV-K elements under study are targeted by TDP-43 in their specific chromatin context within the genome. Expression of HERV-K, notably the env product, either by transfection of the prototypal HERV-K genome or HERV-K env gene into human neuronal cultures, or through transgenic mice expressing HERV-K env gene under the pyramidal neurons expressing Thy-1 promoter at a similar or higher level as observed in ALS patients, all caused neurotoxicity [141], triggering the degeneration of motor neurons and affecting the length, branching, and complexity of the dendrites as well as the number and the morphology of the spines. In the frontal cortex of the transgenic mice, yH2A.X foci were increased in neurons, and astrocytosis was noted in the surroundings, highlighting ongoing neuronal injury [141]. Interestingly, Cas9-directed downregulation of HERV-K env naturally produced in the prostate cancer cells LnCAP can trigger a strong diminution of TDP-43 mRNA and protein levels [142], suggesting the existence of a mutual activation loop between TDP-43 and HERV-K proviruses encoding env ORF. This system could be leveraged to downregulate TDP-43 overexpression. A schematic figure illustrating these findings is presented in Figure 3C. At this stage, however, and as underlined by Douville and colleagues [140], it remains unclear if the recombination of various HERV-K proteins originating from multiple loci may activate cycles of retrotransposition (or reinfection) and result in DNA damage leading to cell death. In addition, the youngest HML-2 family members, i.e., those belonging to the LTR5Hs that hold the activating TDP-43 binding site, present some degree of insertional and structural polymorphism in the population [117,118,119]. Furthermore, besides the potential retroviral ORFs production, LTR5Hs LTR elements have been shown to regulate hundreds of “cellular” genes [143]. These are all aspects of the TDP-43 regulation of the HERV-K family that deserve further investigations with respect to neurodegenerative diseases in which TDP-43 functions and levels are described to be altered.

#### 2.5.2. TDP-43 Control of LINE1 Retrotransposition

Heterochromatic regions are typically enriched for different types of intergenic repeats and depleted from genes. In agreement, the loss of heterochromatin identified in post-mortem human ALS brain nuclei without TDP-43 by Liu and colleagues via ATAC-seq was enriched for a particular class of interspersed intergenic repeat, i.e., the LINE1 elements [65]. Importantly LINE1 decondensation was not only accompanied by an increase in L1 transcription, indicative of their derepression, but also by increased LINE1 DNA in neuronal cells, meaning an increase in the number of LINE1 copies within the genome [65]. Functional LINE1 elements have the capacity to insert neo-retrotranscribed copies of themselves in ectopic places of the genome, a sort of “copy–paste” mechanisms. Hence, the authors of this study demonstrated that nuclear TDP-43-lacking cells displayed an increase in LINE1 retrotransposition [65]. In keeping with this view, in vitro experiments directly correlated the lack of TDP-43 in HeLa cells’ nuclei with a decrease in H3K9me3 histone heterochromatin modification and an increase in retrotransposition activity. This cell population was neuronal and corresponded to 7.05% of them on average and less than 2% of all cells [65].

These results suggest that there may be an accumulation of LINE1 nucleic acids in TDP-43-negative nuclei, which can potentially increase LINE1 DNA content, even in the absence of complete retrotransposition leading to truncated L1 [135]. Increases in L1 and/or in ERV RT are both consistent with the previously reported increase in RT activity in serum of HIV-1-negative ALS patients [144,145]. A new study, this time conducted in the mouse germline, shows that TDP-43 plays also an important role in inhibiting L1 retrotransposition in mouse embryonic stem cells (mESCs) and preimplantation embryos [146]. In this study, it was shown that TDP-43 interaction with L1 open reading frame 1 protein (L1 ORF1p) is necessary in order to mediate this genomic protection. It is important to note that this process is developmentally regulated; L1 retrotransposition is highly active in mammalian pre-implantation embryos [146]. Furthermore, an L1 retrotransposition assay in HEK293T cells revealed that deletion of the TDP-43 C-terminal domain severely compromised the inhibition of L1 retrotransposition, while RRM or NLS mutants retained their inhibitory capacity [146].

Therefore, in the brain of ALS patients, TDP-43 alterations may lead to LINE-1 reactivation via H3K9me3 reduction and chromatin decondensation, but in some instances, they could also be directly linked to the increase in L1 new copies integrated into the neurons genome. It is interesting to stress that in another pathological condition, i.e., a mouse model of progeria, *L1* RNA was shown to negatively regulate the enzymatic activity of the H3K9me3 SUV39H1, thus sustaining heterochromatin loss [147]. A schematic illustration of these findings is presented in Figure 3E. 

#### 2.5.3. Conservation of TDP-43/TDPH Regulation of TE in Drosophila

Flies have often represented a good model for TDP-43 deregulation and ALS. For this reason, TDP-43 impact on TE was recently examined ([148,149,150]. While the ERV-K family is not present in Drosophila, hTDP-43 overexpression in different brain cells of Drosophila (including neurons and glia) affected the expression of TE elements, principally the LTR and LINE classes, and generally triggered their activation [148,149]. The same was observed following TBPH loss in TBPH-null fly head tissues (TBPH being the homologue of TDP-43 in flies) [150]. Specifically in glial cells, this led to the activation and to the replication of ERV-related Gypsy retrotransposon, which appeared to be responsible for a substantial portion of the toxicity observed upon hTDP-43 overexpression [148,149,150]. Notably, both non-cell-autonomous propagation of DNA damage and apoptosis experienced by the adjacent neurons could be blocked by the Gypsy ERV glial silencing [148,149]. When investigating the siRNA pathways, which is a well-known mechanism of TE silencing, researchers noted that hTDP-43 expression interfered with siRNA-mediated—but not the miRNA-mediated—silencing, resulting in the desuppression of a reporter expression [148]. In glial cells, the reduction in siRNA silencing efficacy was marked and rapid, while in neurons, it was progressive and age-dependent [148]. Again, similar findings were obtained in TBPH-null Drosophila [150], and it was further found that TBPH interacts with the RISC component *Dcr*-2 mRNA and protein, regulating both its levels and activity [150]. This indicated an additional mechanism by which TDP-43 pathology could lead to TE silencing erosion and genome instability, including TE-mediated DNA damage. A schematic illustration of these findings is presented on Figure 3D.

Although, siRNAs and miRNAs in Drosophila are processed largely via distinct pathways—Dcr2/Ago2 and Dcr-1/Ago, respectively—in mammals, the same DICER and Argonaute proteins process both miRNAs and siRNAs [151], a process in which TDP-43 has been shown to be implicated for at least a subset of miRNAs (see the review of [152]). In humans, suppression of TDP-43 in the neuroblastoma SH-SY5Y cells was found to produce a similar reduction in the human Dicer protein levels [150]. Furthermore, in the germ line, another type of siRNA using the ping-pong pathway (piwiRNAs and miwiRNAs) in mice and Drosophila derives from a large group of retrotransposons, which themselves and are linked to retrotransposon silencing and DNA methylation [153]. Similar mechanisms using the endo-siRNA pathways can drive LINE-1 DNA re-methylation in human breast cancer cells [154].

Interestingly, reverse-transcriptase inhibitors alone (stavudine, azidotimidine, tenofovir, or rilpivirine) has also been demonstrated to be effective in partially reverting the locomotion defects in TDPH-deficient flies induced by RTEs activation, with azidotimidine been the most efficient. Enotaxin, a compound capable of activating the siRNA pathway and able to counteract RTE activation, was also able to restore the locomotive behaviors and the formation of neuromuscular synapsis [150].

### 2.6. ALS and Epigenetic Functionality of TDP-43 Short Splicing Isoforms

**Figure 4:**
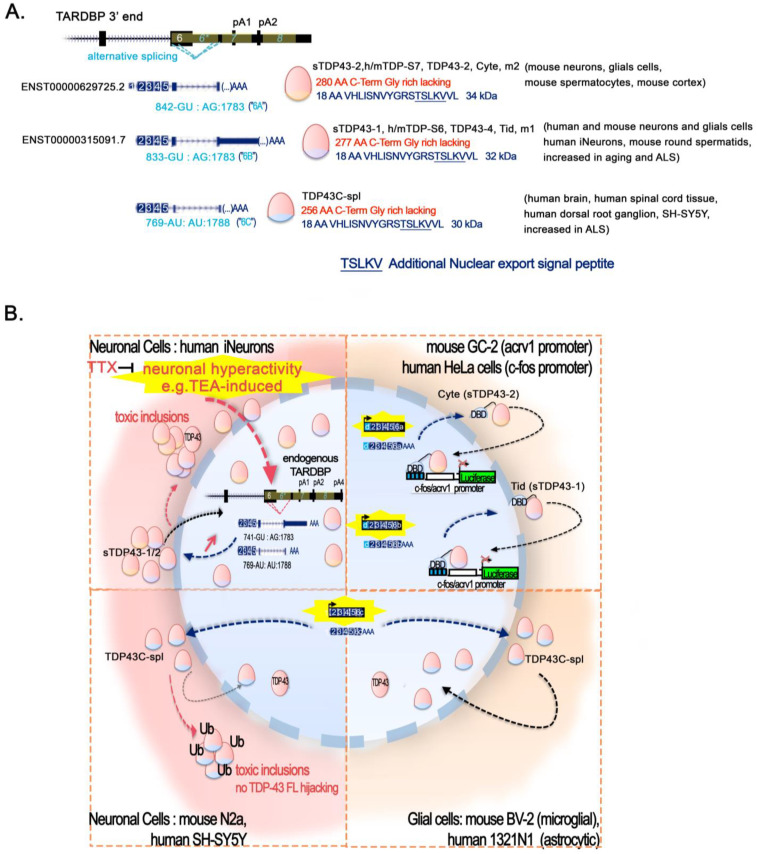
Short-TDP-43 isoforms: alternative splicing and context-specific fate. (**A**) Alternative splicing leading to short-TDP-43 proteins in brain. Transcripts resulting from various splicing of the alternative intron 6 (*) within TDP-43 pre-mRNA and translated into different short-TDP-43 proteins readily observed in mouse and human brains are depicted: ENST00000629725.2 encoding sTDP43-2 (also called hTDP-S7, mTDP-S7, TDP43-2, m2, or Cyte); ENST00000315091.7 encoding sTDP3-1 (also called hTDP-S6, mTDP-S6, TDP43-4, m1, or Tid). Both are highly conserved at the transcript and protein levels and the recently identified transcript producing TDP43C-spl. Note that the TSS and the TTS of the transcripts of these isoforms have not been validated, and it is not clear if they hold the 3′UTR TDPBR needed for autoregulation via TDP-43 FL. Short TDP43 isoforms-specific intron borders with splice donors (SD) and acceptors (SA) are indicated under each transcript in blue, with numbering given relative to the CDS +1. SD and SA are all located within TARDBP exon 6, eliminating the majority of exon 6. The sTDP43 protein isoforms contain at least the first 256AA and up to the first 280AA of TDP-43 (indicated in red). They contain the N-Term NLS, the RRM 1 and 2, and the NES, but not the C-terminal glycine-rich region in the TDP-43 protein. They gain an alternative 16 to 18AA, forming a unique C-end term VHLISNVYGRSTSLKVV, and sheltering a second nuclear export sequence (NSE) consisting in TSLKV. sTDP43-1, sTDP43-2, and TDP43C-spl have a mass of about 34 kDa, 32 kDa, and 30 kDa, respectively, and have been spotted in several vulnerable zones of the CNS both in normal and ALS patients, as well as in mouse male germ cells, as indicated between brackets. (**B**) Cell type sensitivity to high expression of short TDP proteins. Abnormal localization and ubiquitination of short TDP-43 aggregates leading to toxic inclusions are a pathological hallmark of neurons and glia in neurodegenerative diseases. Note the repressive potential of the mouse short TDP-43 isoforms Cyte and Tid (sTDP43-2 and sTDP43-1, respectively) on *Acrv1* and *c-fos* promoters in GC-2 and Hela cells. The tethering of Cyte or Tid to a reporter plasmid using Gal4 DNA binding domain (DBD) fused to TDP-43 at Gal4 binding sequences (blue boxes) represses *c-fos* or *Acrv1* promoters-induced luciferase expression, as does TDP-43 FL in the same conditions (up right frame). In neurons (left frames), human and mouse sTDP43-1 and 2 are present either in the nucleus or in the cytoplasm (soma and axons) or in both. They are upregulated by age and in response to increased neuronal activity (e.g., TEA in human iNeurons, or bicuculline in rodent primary mixed cortical neurons), and are conversely downregulated by TTX that abolishes neuronal activity. When overexpressed, they form insoluble aggregates in the cytoplasm able to sequester full-length TDP-43, leading to nuclear clearance of endogenous TDP-43 and neurotoxicity. TDP-43C-spl is observed in the cytoplasm of the human spinal cord, brain tissue, and dorsal root ganglia. TDP-43Cspl overexpression in neuronal cell lines convey their delocalization to the cytoplasm, where they form toxic ubiquitinated aggregates. In astrocytoma and microglia cell lines, TDP-43Cspl is not delocalized to the cytoplasm and localizes in interchromatin granule clusters (speckles) in the nucleus. TDP-43 is not recruited to the TDP43C-spl aggregates, but keeps its nuclear localization (bottom right frame).

Naturally occurring splice variant isoforms of TDP-43 leading to shorter TDP-43 proteins have been revealed on several occasions since the early 2000s [156,157], but their possible relevance in ALS has just started to be investigated. They all derive from the different use of close splice donors/acceptors sites within the exon 6 in the 3′ end of the TDP-43 ORF and part of the 3′UTR, creating a sixth intron (Figure 4A). The resulting proteins share at least AA1-AA256 with TDP-43, but they have an alternative C-terminus, lacking the highly disordered Glycine-rich region, and gaining an additional 18 AA sequence at the C-terminal end that is not present in wild-type TDP-43. 

For example, in the study on the *Acrv1* gene regulation by TDP-43, the author identified several RNA isoforms of mTDP-43 in testicular tissue [40]. These TDP-43 splice variants, Cyte and Tid, were cloned from mouse spermatocytes and round spermatids, respectively. The spermatocyte splice variant contains three amino acids more than the round spermatid variant at position 278–280 [40]. When produced by a vector to be driven in close proximity of a reporter-plasmid-holding *Acrv1* promoter in GC-2 cells or minimal *c-fos* promoter in HeLa cells, these variants were able, like the TDP-43 FL, to repress their expression [40] (Figure 4B). These short isoforms have been repeatedly identified in neurons (sTDP-43-1 and sTDP-43-2) from mouse and human, where they appear to be either nuclear, cytoplasmic, or both, and were shown to be upregulated by neuronal hyperactivity [158,159]. In this case, the short isoforms were observed to accumulate in the cytoplasm, where they formed insoluble inclusions and sequestered the full-length TDP-43, possibly via preserved N-terminal interactions [159,160] with toxic consequence for the neurons (Figure 4B). Importantly, both the transcripts and proteins related to sTDP-43-1 and sTDP-43-2 (at least) are highly conserved in humans, non-human primates, and lesser mammals [157,159]. In particular, both in humans and mice, sTDP-43 transcripts were found enriched in vulnerable motor neurons, and neurons and glia of ALS patients are marked by a striking accumulation of sTDP-43 [159]. Intriguingly, the same unique C-terminal 18 AA, which is included in these isoform, contains an additional, unique, NES sequence (TSLKV) to which has been attributed a strong bias for cytoplasmic localization [159]. sTDP43 species in the neurons from five patients with neurodegenerative diseases (MSA and DLB with AD, C9ALS, and sALS) were either cytoplasmic only or cytoplasmic and nuclear in the case of full-length TDP-43 being mislocalized to the cytoplasm. When full-length TDP-43 was still nuclear, sTDP43 proteins appeared also to be nuclear-only in most cases, or else were cytoplasmic only, perhaps representing, in this case, an early stage of the pathology [159]. In any case, keeping in mind that not all cells expressed sTDP-43s, when expressed, sTDP43 species had nuclear localization in an abundant proportion of neurons.

These protein variants appear to be defective for splicing and for the regulation of full-length TDP-43 through the autoregulation mechanism (see dedicated chapter), a result that is in accordance with the lack of the glycine reach C-term region [159]. Their presence and function in the nucleus is thus puzzling. However, considering the chromatin association of these forms reported in male germ cells and their capacity to repress different promoters in vitro [40], their function could be related to chromatin and transcriptional regulation. They could thus be modulated according to the cell types, differentiation state, or activity, and could functionally overlap, complement, or compete with TDP-43 in its DNA-related regulatory attributes. In support of this, an additional novel spliced isoform, with an alternative 16AA C-term holding the TSLKV NES and TDP-43C-spl has been reported in a very recent study [160]. This new isoform is expressed in the human spinal cord, brain tissue, and dorsal root ganglia. Upon overexpression, this isoform seems to harbor a cell-type dependency for the formation of cytoplasmic ubiquitinated aggregates in neuronal cell lines. In astrocytoma or microglial cell lines, it localizes in the nuclear space forming speckles [160]. Finally, when forming inclusions in the cytoplasm, these isoform aggregates do not contain full-length TDP-43 [160], contrary to what has been observed with sTDP43-1/2 [159]. A schematic illustration of these findings is presented on Figure 4B.

### 2.7. Epigenetic Role of TDP-43 Alternative Forms

Another intriguing finding of the work performed by Giannini and colleagues is the presence of the TDP-35 form at chromatin at R-loop, as highlighted by co-IP in both whole-cell lysates and chromatin fractions of lymphoblastoid cell lines (LCL). Regarding the importance of TDP-43 disease-associated mutations, it is interesting to note that this interaction was higher in for LCL carrying the A382T mutation of TDP-43 [97].

The authors initially understood TDP-35 to correspond to CTF35, a C-terminal fragment of 35 kDA, resulting from the truncation TDP-43 [97] using an antibody against AA203-209 [161]. However, the caspase induction of TDP-43 into cytotoxic CTF-35 is generally known to happen in the cytoplasm and to accumulate in detergent insoluble fraction. Furthermore, caspase-generated CTF-35 fragments have a disrupted nuclear localization signal (NLS), making them unlikely to travel back to the nucleus. In addition to these events, shorter-than-35-kDa TDP-43 immunoreactive products are numerous and can result from cleavage by other enzymes or potentially derive from alternate ORFs. In particular, analysis of Neuro2a cell lysates evidenced that Ca^2+^-activated calpain cleavage produces N-term fragments identified to be of about 36, 34, and 32 kDa [162]. Another work comparing caspase- and calpain-generated TDP-43 fragments via in vitro protease digestion of produced full-length TDP-43 shows a more complex pattern of cleavage product [163]. Notably, Caspase-3 induced 33 kDa fragments, whereas calpain produced 35 kDa fragments, and both could lead to the generation of 25 kDa fragments. Accordingly, calpain-I and caspase-3 cleavages have been shown to lead to several fragments being recognized exclusively by antibodies raised against either the N-term, the C-term, or internal epitopes [162,163]. This complexity highlights the difficulties in interpreting the biology of TDP-43 regulation and function, and the crucial importance of the tools used to identify TDP-43 fragments, as already reported. Notwithstanding these challenges, the results presented in both studies have highlighted the possibility that the chromatin associated fragment identified by Giannini and colleagues [97] could be an NTF of about 35 kDa rather than the thoroughly described CTF35. On top of this, another recent work has described TDP35 in the nucleus, detected using a C-term antibody (Gly 400 epitope). The authors of this work reported that this CTF35 was produced by the activity of caspase3 on TDP-43 in the nucleus, and that this proteolytic cleavage could be impeded by *Malat-1* lncRNA binding to TDP-43 in the nucleus [164]. In any case, the fragment observed by Giannini and colleagues has been reported to exist in association with the RNA–DNA hybrid (detected by S9.6 antibody) on the chromatin, as well as in Neuro2a cells in the absence of calpain-I treatment, along with the 35 kDa TDP-43- related products [162]. Finally, in addition to all these species, it has been postulated that human and mouse cortices also show reactivity for N-term TDP-43 antibodies at 32.5 kDa and about 35 kDa of size, possibly corresponding to sTDP43-1 and sTDP43-2, fragments upregulated by neuronal hyperactivity [158,159] or to the recently identified sTDP43C-spl [160]. Indeed, all these alternatively spliced isoforms, as mentioned in the previous (Section 2.6: ALS and Epigenetic Functionality of TDP-43 Short Splicing Isoforms) section, have been shown to localize in the cytoplasm or in the nuclear space either on chromatin or in speckles, depending on the various experimental conditions. Regardless of the effective(s) scenario, the presence of short TPD-43 products—either splicing isoforms or proteolytic cleavage-products of full-length TDP-43—in the nucleus and, in particular, on the chromatin, will certainly require further investigation pertaining to their functional output in physiological and pathological situations.

### 2.8. Epigenetic Landscape Modifications Associated with TDP-43 Mutants

TARDBP mutations have been identified mostly in familial ALS patients but also in sporadic FTD, AD, and PD cases, and more than 50 TDP-43 variants have been linked to the incidence of ALS/FTD [165,166]. They are mostly found in the C-term part of the protein, while in the N-term, A90V in the NLS and P112H and D169G have been reported in the RRM1 motif [165]. Functional observations have been made for about twenty of ALS-mutants, as reviewed in [165,166] and for mouse and in vitro models holding TDP-43 mutations, as described in [167,168,169], reporting altered normal RNA splicing with or without concomitant cytoplasmic aggregation.

Few TDP-43 models carrying null or point mutations have been investigated to explore the association of pathological TDP with altered epigenetics treats. We have reported them in the previous sections in relation to their impact on chromatin in their dedicated sections. In the present paragraph, we condense the impact of TDP-43 mutations on chromatin homeostasis from a mutation point of view. 

Depending on the regions hinted by mutations, different scenarios on chromatin impact can be envisaged. Indeed, mutations in the C-term—known to guide nuclear loss—are expected to have a broad impact on TDP-43 functions both at the RNA and the DNA levels. This might naturally hold true for mutations within the NLS or the NES controlling the nucleocytoplasmic shuttling. Instead, mutations in the N-term, whether at the RRM1 or RRM2, may have a more focalized impact on the RNA-processing aspect or on chromatin, whether linked to RNA processing aspects or lncRNA-related functions of TDP-43 on chromatin, but also on TDP-43′s ability to bind ssDNA or dsDNA, as has been documented in different studies [15,17,19,20,21,22], and notably regarding free dsDNA ends [23]. Such mutations might impact either transcriptional regulation, the ability to bind DNA damaged ends, or the ability to restrict TE activity. For example, mutations of the TDP-43 RRM1 that abolish its DNA/RNA binding, as shown with F147/149L, can be expected to modify TDP-43-mediated gene expression. Consistently, as reported above, mutants either lacking RRM1 motif or with a mutated form (F147L/F149L) are sufficient to disrupt TDP-43 repressive function on the *Acrv1* promoter [40] and on its own promoter [170]. In addition, RRM1–RRM2 acetylation-mimic point mutations (KK-QQ), but not acetylation-null (KK-AA), abolished *CHOP* transcriptional activation [48]. Instead, deletion of the TDP-43 C-terminal domain severely compromised the inhibition of L1 retrotransposition, while RRM or NLS mutants maintained their inhibitory capacity [141]. Expression of hTDP-43 carrying a mutated nuclear localization signal (ΔNLS-hTDP-43; [63,64]) conveyed notable changes in gene expression, including a dysregulation of histone 3′ end-processing machinery paralleled by an increased canonical histone transcript, reinforcing the remarkable role that TDP-43 has in the function of the chromatin assembly pathway.

All ALS/FTD TDP-43 mutations reported to date to have an impact on chromatin are concentrated in the C-term region and lead to either a defect in DNA methylation or DNA damage or to both, and some also impact TDP-43 own regulation (Table 2).

Finally, as we detail further in the next section (Section 3: TARDBP/TDP-43 Autonomous/Non-autonomous Regulation), some mutations identified in ALS patients by Luquin and colleagues [172] in the promoter region of TARDBP promoter have been tested in different cell lines without significant effect on its activity in the tested conditions [173].

## 3. TARDBP/TDP-43 Autonomous/Non-autonomous Regulation

**Figure 5:**
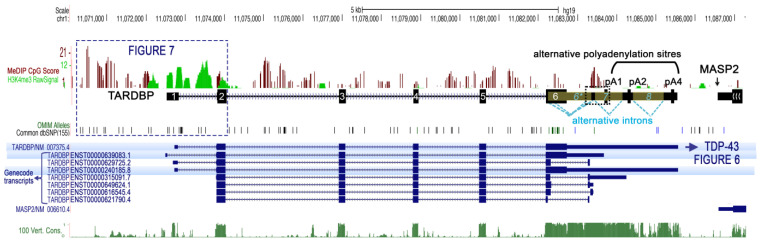
Human TARDBP locus organization. Human TARDBP locus (black track; NCBI Gene ID: 23435), encoding full-length TDP-43 (FL) protein, is localized on chromosome 1p36.22 upstream of the MASP2 gene locus and counts up to 10 exons (obligatory and alternative exons). The blue tracks report the reference transcript coding for TDP-43 FL (NM_007375.4), as well as other predicted alternative transcripts isoforms (GENCODE. Version 42lift37 (Ensembl 108), ENST00000639083.1, ENST00000629725.2, ENST00000240185.8, ENST00000315091.7, ENST00000649624.1, ENST00000616545.4, and ENST00000621790.4,). NM_007375.4 is composed of six exons, flanked by a 5′ and a 3′ UTR. The 3′UTR embedded in exon 6 also contains two cryptic introns, intron 6* holding multiple alternative splicing sites and intron 7 (yellow frames, with splicing highlighted with blue dashed lines), as well as alternative polyadenylation signals (PAS) that have a fundamental importance for TDP-43 production control. The bottom dark green track shows the conservation within 100 vertebrates (“100 Vertebrates Conservation by PhastCons”). On the top of the TARDBP locus UCSC genome browser, tracks are reported. CpG score (red bars) shows different degrees of CpG methylation along the locus as found in the human cortex. H3K4me3 histone modification (green bars), the enrichment of which marks active promoter regions, is found upstream of exon 1 (TSS) down to exon 2. Common SNP (black and blue bars; from update V155) are found mainly within introns. OMIM variants (in green bars) are mainly in the exon 6, containing both the DNA and RNA signals for auto-regulation, and the region coding the C-term of the TDP-45 protein. All tracks are from the UCSC Genome Browser on Human (GRCh37/hg19).

In this section, we present the existing knowledge regarding TDP-43 and, more generally, TARDBP locus regulation. A schematic diagram of the TARDBP locus structure is presented in Figure 5.

### 3.1. TDP-43 Autoregulation

**Figure 6:**
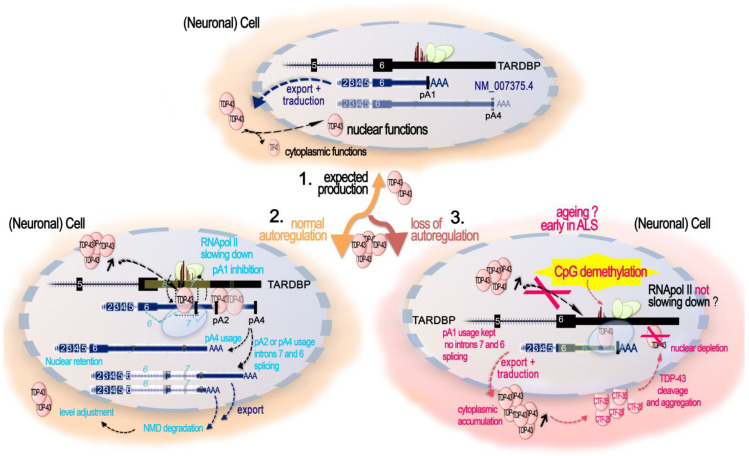
Role of DNA CpG methylation in TDP-43 levels autoregulation. 1. Expected TDP-43 production. TDP-43 production is promoted by the transcription of an mRNA (blue, NM_007375.4) with 6 exons and usage of poly(A) signal (pA)1 located just after the TDPBR both within the alternative intron 7 (yellow box ≪7≫) or extending up to the pA4. When present at adequate levels, the mRNA is exported to the cytoplasm and is translated to produce TDP-43. 2. Normal autoregulation. When nuclear levels of TDP-43 increase over the expected threshold, TDP-43 binds co-transcriptionally to its own pre-mRNA at TDPBR and down to pA1, stalling RNA pol II at DNA in the correspondence of this region. This impedes pA1 signal usage and facilitates the splicing of the cryptic intron 7. At this stage, three scenario exists: (**1**) alternative use of pA4 generates an mRNA, mainly retained in the nucleus, with no additional splicing and holding a long 3′UTR; (**2**,**3**) pA2 or pA4 are used, and the alternative splicing of cryptic intron 7 and then 6 are triggered. The double-spliced isoforms are exported to the cytoplasm and become degraded via nonsense-mediated mRNA decay. These autoregulatory mechanisms, by resulting in a decrement in the cytoplasmic TDP-43 FL mRNA, allow for the adjusting of the TDP-43 protein levels. 3. Autoregulation failure and role of CpG methylation in the 3′UTR DNA region. The amount of TDP-43 in the nucleus determines the ratio of these isoforms, and when nuclear TDP-43 levels are reduced, the splicing is repressed. The absence of nuclear TDP-43 induces an abnormal autoregulation and increases the amount of TARDBP mRNA in the cytoplasm. The DNA region of the 3′UTR spanning the alternative exon 6-exon 7 junction in correspondence to the TDPBR on pre-mRNA bears a track of 15 CpGs, methylated in the human cortex. When these 15 CpGs are demethylated, TDP-43 autoregulation is negatively affected, suppressing the alternative exon 7 splicing and increasing canonical mRNA levels. The precise mechanisms are not known but possibly involve a reduction in RNA Pol II pausing or alteration in the recruitment of other factors. In vitro, this region is sensible to Tet1-induced demethylation and DNMT3b-induced re-methylation, both of which modulate intron splicing. In the human control motor cortex, DNA methylation at 3′UTR CpGs 10–15 is inversely correlated with age, and, generally, the CNS appears more affected than the liver, showing disparities among regions, with the motor cortex having the lowest CpG 10-15 methylation levels.

In normal conditions, TDP-43 can regulate its own protein levels through a negative nuclear feedback loop triggered by binding to its own RNA in the 3′UTR region. This causes additional splicing of *TDP-43* mRNA, its nuclear retention and its decay, and, subsequently, a decrease in TDP-43 protein production [34,174,175,176] (Figure 6). This process appears to take place co-transcriptionally and involves the corresponding gDNA sequence. More precisely, when overexpressed, TDP-43 binds to a cryptic intron within its 3′UTR (TDPBR, TDP Binding Region), stalling the RNA polymerase II within this TARDBP 3′UTR gDNA region, promoting intron 7 alternative splicing and use of downstream polyadenylation sites. This event gives rise to the use of a sub-optimal pA2 site and a longer transcript isoform that is retained within the nucleus and subsequently degraded [34,174,175]. Like intron 7, intron 6 undergoes alternative splicing as well, and this was shown to play an important role in autoregulation, notably for ALS [177]. In this study, it was shown that inhibiting the splicing of cryptic intron 6 using antisense oligonucleotides (ASOs) in vivo in mouse or in vitro in human IPSC-derived neurons could increase *Tardbp* mRNA expression, and this either increased the amount of fragmented insoluble TDP-43 or decreased TDP-43 nuclear amount. In addition, the number of motor neurons in the mouse spinal cord was reduced. These non-genetically modified models emphasize the importance of TDP-43 autoregulation turbulence for the pathogenesis of ALS [178]. Indeed, it has been proposed that TDP-43 pathology involves a vicious cycle in which excessive TDP-43 is continuously produced as a result of a defect in the autoregulatory mechanism [177,179].

Concerning ALS-FTD with TDP-43-mutations, two mouse models of ALS-FTD holding either the Q331K or the M323K mutation in the endogenous TARDBP obtained by knock-in showed a perturbed autoregulation [167,168]. Q331K mutant mice had an increase in *tardbp* RNA and TDP-43 nuclear protein in the frontal cortex and spinal cords, but not in their motor neurons [168]. M323K knock-in mice also had an increase in *tardbp* RNA level, but the increase was not significant for TDP-43 protein [167]. Interestingly, the mutation leading to the Q331K on the protein is located in the cryptic intron 6, as are many other mutations, and is shown to affect cryptic intron 7 splicing [168]. It is not clear, however, if the mutation can also affect the protein function itself, or some unknown splicing regulation signal within the intron 6 at the RNA or the gDNA level, and both possibilities are not mutually exclusive. In any case, it is clear that transgenic mice overexpressing human TDP-43 with the Q331K mutation show a reduction in the endogenous *Tardbp* level, attributed to classical autoregulation [169]. Interestingly, post-mortem brain tissues from ALS patients TDP-43-negative nuclei in which L1 decondensation has been reported were also shown to specifically display a reduced autoregulation, indicated by decreased splicing of the cryptic 3′UTR intron with respect to TDP-43-positive nuclei within the same brain region. In addition, a reduced overall TARDBP gene expression defined by a significant reduction in *TARDBP* reads was observed in TDP-negative cells with respect to the paired TDP-43-positive neurons [65]. This is a quite surprising finding that could indicate that auto-regulation can also takes place at the promoter region. Alternatively, this reduced TDP-43 transcription could be caused by neuron cell-type identity differences between TDP-positive and TDP-negative pools, considering that markers of superficial neocortical neurons were significantly enriched in TDP-negative cells [65].

From this review point of view, it is nonetheless important to highlight the recent findings from Koike and colleagues [180], showing that control of splicing-linked autoregulation is also governed by epigenetic mechanisms and at least by DNA methylation of the TARDBP region critical for autoregulation (Figure 6). In fact, the 3′UTR region of the TARDBP locus contains a set of 15 CpGs around and downstream of the intron 7 5′ splice donor site that are normally methylated in the human cortex [180]. The importance of DNA methylation controlling this process was shown using the dCas9 system to selectively demethylate this specific CpG region by the demethylating protein TET1, demonstrating that demethylation of the CpGs 10–15 suppresses the alternative splicing of intron 6 and intron 7 and increases the levels of *TARDBP* mRNA. In the human motor cortex, normal aging was associated with a decrease in the methylation of this region and its degree correlated with *TARDBP* mRNA levels. However, an accelerated DNA methylation age in the ALS motor cortex was associated with a younger age of onset, suggesting its potential involvement in ALS pathogenesis [180].

### 3.2. Production of Alternatively Spliced Protein-Coding Isoforms

It is widely acknowledged that TARDBP locus presents a complex architecture associated with the production of several alternative splicing isoforms, some of which neither block translation nor lead to nonsense-mediated decay, but produce alternative functional TDP-43 proteins. Indeed, alternative splicing in exon 6 can lead to shorter proteins, identical to TDP-43 from AA1 to AA256 at least, but lacking the disordered Gly-rich region. As a result, these isoforms end with alternative AA stretches in the C-terminal region able to influence their nucleocytoplasmic shuttling and creating a new nuclear export signal (NES) with different susceptibility according to the cell type and/or differentiation stage [40,158,159,160] and probably to modulate the function and/or the DNA/RNA targets attributed to TDP-43 FL. Some of these isoforms appear to be upregulated by neuronal hyperactivity and to drive TDP-43 pathology in ALS [159,160], as for the TDP-43 FL transcript [159], indicating a possible general induction of the TARDBP locus. In *C. elegans*, the relative abundance of two rare splice junctions of TDP-43 homolog tdp-1 can be regulated by SUP-46 [181]. In particular, the rare junctions between exons 3 and 5 and between exons 5 and 6 are, respectively, decreased and increased in mutant sup-46(qa710) [181]. SUP-46 is an RNA binding protein from the HNRNP-M family. Its human homologs are HNRNPM and MYEF2, and are necessary for the response to acute and chronic heat stress. Additionally, in human HEK293 as in worms, SUP-46/HNRNP-M and, to a lesser degree, MYEF2 were shown to interact with TDP-43/TDP-1 and to co-localized in somatic nuclei [181,182]. These findings could indicate SUP-46/HNRNP-M as another TDP-43 partner in TDP-43/TARDBP physiopathological regulation. In general, however, the mechanisms and factors that dictate/favor the production of these isoforms and how they impact regulatory function of TDP-43 are poorly known yet. 

One thing that can be said, however, is that the TDP-43 isoforms appear comparatively much less expressed than TDP-43 FL mRNA in human iNeurons and WT mouse cortex [159]. However, their relative balance appears to be a matter of tissue and of aging, as shown in the study of Weskamp and colleagues [159].

Thus, in mouse, compared to frontal cortex homogenate where they contribute around 1% of *TARDBP* transcripts, sTDP43-1 and sTDP43-2 splice events were found to be highly enriched in lumbar motor neurons, reaching 17% and 22% of total *TARDBP* transcripts, respectively. In the human, by re-examining available RNA-seq data, the authors consistently observed significant expression of sTDP43-1 but not sTDP43-2 in several different regions of the CNS, including but not limited to the spinal cord ventral horn, spinal motor neurons, cerebellum, and frontal cortex, making from 30% up to 55% of the TARBDP transcripts. In the lumbar motor neurons, sTDP43-1 even reached almost 70% of the total transcripts against less than 10% of TDP-43 mRNA. Lumbar glial cells also contained significant amounts of the isoform proteins. No significant differences between control, sALS, and C9ALS (cerebellum and frontal cortex) patient samples were identified [159]. These data show that a large and highly variable contribution of sTDP43-1 is human CNS, at least in adult post-mortem tissues. This phenomenon could be linked to aging and contribute to ALS. Indeed, WT mouse display a significant age-related decrease in TDP-43 FL mRNA and increase in sTDP-43-1 and sTDP-43-2 transcripts abundance. Notably, these levels are not affected by the TDP-43 Q331K mutation [159]. In addition this could be linked to the difference in these transcripts to be autoregulated in the same way as the TDP-43 FL mRNA. Actually, from the study of D’Alton, Altshuler, and Lewis, TDP-43 autoregulatory capacity does not extend to all transcripts, but seemed restricted to endogenous TARDBP transcript encoding TDP-43 FL [158]. As for their apparent association with aging and ALS, a deeper understanding of the mechanisms and factors contributing to regulating their production would be important for the field of TDP-43 proteinopathies.

### 3.3. Promoter Control of Pan TARDBP Expression

Although TDP-43 is ubiquitously expressed and can regulate/maintain its own expression through the negative feedback loop discussed above, some observations suggest that its overall expression level is not frozen but can also experience regulated variations in time and space. In particular, each tissue has its specific amount of TDP-43, and these are adequately compensated in Tardbp +/− mice in physiological condition [75]. In addition, TDP-43 levels seem to be developmentally regulated, and a age-related decrease in its expression has been noted in a variety of organisms such as fruit flies [183] and mice [184,185,186]. 

Thus, regulation of its expression might also occur at a more classical transcriptional level through its promoter. While the mechanisms of it auto-regulation by splicing and NMD decay have been the focus of many efforts, the promoter features have been less intensively investigated until the last two years [170,172,173,186]. We review here these new findings, including genetic and epigenetic characteristics.

**Figure 7:**
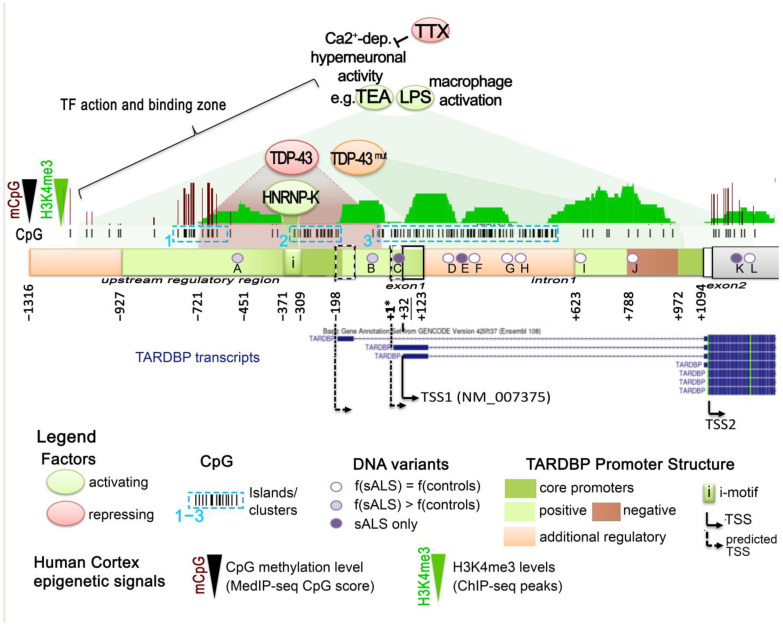
TARDBP locus promoter regulation. The main TSS identified for TARDBP and TDP-43 FL is from Seq NM_007375.4 (TSS1), starting at chr1; 11072711(+strand) (GRCh37/hg19). It is located in exon1 (Ex1), 102bp before intron1. Other confirmed (solid arrows, TSS2) or predicted (dashed arrows) TSSs lay upstream or downstream of TSS1 and are displayed. A minor TSS (TSS2) within exon2 (Ex2) has been identified in human fetal brain, without a precise localisation reported. Note that since the predicted TSSs at −32 served as a reference for start-numbering in different works, it is indicated here as ≪+1*≫, representing a shift of −32 bp relative to TDP−43 FL Seq transcript NM_007375.4 TSS (TSS1). The TARDBP promoter region is bipartite, with two core promoters (dark green boxes): one proximal TATA-less promoter located before the exon1 (−327–+1) and within (−451–−230); and one distal located at +972_+1094, i.e., within the intron1 position +850_+972. The proximal TATA-less promoter is necessary for the minimal promoter activity in all tested cell lines. Other regulatory features identified are positive (green boxes) and negative (red boxes) regulatory regions. A 58 bp region (−281–−223) in the proximal core is crucial for promoter activity. The upstream regulatory region (−927–−300), holding an iMotif (−371–−309), is important for maximal activity. Sequences in the +1–+123 region, encompassing exon1, positively enhance transcription. In the distal intron1 promoter, the region +788–+972 (+666–+850 of intron 1) is repressive. Epigenetic characteristics of TARDBP locus in the human cortex are displayed, as in Figure 5. H3K4me3 histone modification (green bars), the enrichment of which marks the active promoter regions and is found upstream of exon 1 (TSS) down to exon 2. The promoter region extending from −836 to + 1106 contains 125 CpG mostly within 3 CpG islands (CGI, dashed blue boxes) (−874 to + 1069). In the human cortex, along the promoter region, the CpG score (red bars) shows different degrees of CpG methylation. The region from CGI 2–3 down to exon 2 is not methylated. TF action and binding zone: factors shown to activate or repress TARDBP promoter are displayed on top of the locus by green or red zone, respectively. LPS and neuronal hyperactivity (triggered, e.g., by TEA, inhibited by TTX) positively regulate the TARDBP promoter, although the specific regions have not been defined yet. TDP-43 itself can repress its cognate promoter and, in particular, through the proximal upstream promoter. TDP-43mut: two TDP-43 mutants (G348C, A382T) activate the intron1 distal promoter. HNRNP-K binds to the iMotif in the upstream tropism. ALS-linked variants: position of variants identified in ALS patients, with their non-different higher frequency (f(ALS)) relative to frequency in HapMap controls (f(controls)), or found in ALS only, are depicted in white-to-purple circles within the TARDBP regulatory region. A (c.1-562t>c, rs9430335); B (c.1-100t>c; rs968545); C (c.13g>a); D (c.122+85c>t); E (c.122+95c>t); F (c.122+150delg); G (c.122+218c>t); H (c.122+284g>t); I (c.123-450a>c); J (c.123-262-263del); K (c.170c>t (p.N12N)); L (c.198t>c (p.A66A)) (see [175]). None of these variants were found to have a significant incidence on the TARDBP promoter in the tested conditions, and no OMIM variant is described in this region to date. All tracks are from UCSC Genome Browser on the human (GRCh37/hg19).

In 2009, prediction of the TARDBP promoter identified a bipartite promoter made from the about 500 bp before the exon1 TSS and two regions within the intron1 [172]. From a mechanistic point of view, in silico analysis and luciferase assays using several constructs have all now confirmed the importance of intron 1 in addition to the region lying upstream of the TSS to promote and regulate transcription [170,173], whereas intron 3 does not display promoter activity [170]. A summary of the findings on TARDBP promoter structure and regulation is presented Figure 7.

ChIP-seq tracks from the UCSC Genome Browser show that the histone mark H3K4me3, the enrichment of which marks active promoter regions, is indeed found from upstream exon 1 (TSS) down to exon 2 in the human cortex [187] and in other cell lines (ENCODE project [188]). Enrichment of H3K4me1 (found near regulatory elements) and of H3K27ac (marking active regulatory elements) in these regions is also observed, further supporting the regulatory role of these regions [170].

The TSS of TDP-43 FL NM_007374.4 results as the main TSS (BestRefSeq, USCS; and [173]), but additional predicted TSSs are located 32 bp and 230 bp upstream of the principal one, and some transcripts in a minor population of TDP-43 transcripts have been reported to start at a TSS within exon 2 in the human fetal brain [170]. The TSS located 32 bp upstream NM_007374.4 has been the mainly used reference start site for the promoter analyses, and numbering in this review kept the same reference unless specifically mentioned.

ChIP analyses in HEK293T cells revealed that TDP-43 binds to its own upstream-and intron1 bipartite promoter [170]. Specifically, luciferase assays showed that TDP-43 could act as a dose-dependent auto-repressor. It negatively impacted the upstream promoter activity spanning the −721 to +1, [170], although such an effect of TDP-43 was not found in the work by Baralle and Romano [173]. In addition, mutation of the TDP-43 RRM1, which abolishes its DNA/RNA binding (F147/149L), was sufficient to disrupt TDP-43 repressive function [170]. Importantly, several ALS-linked mutants of TDP-43 (G348C, A382T) were less efficient in repressing the upstream-intron1 promoter region despite still being able to bind it [170]. Considering that these two mutants tend to induce comparatively early onset of fALS, it is interesting to note that A382T was able to induce a significant activation of the intron 1 promoter. In conclusion, it would appear that TDP-43 not only acts on its mRNA to regulate its expression; it can also act on its own promoter as a transcriptional inhibitor. As a result, TDP-43 disease-associated mutants may contribute to maintaining aberrantly high levels of TDP-43 transcription and thus contribute to ALS early onset and progression [170].

In parallel, hnRNP-K, another hnRNP, was shown to bind to the TARDBP promoter, most probably at its predicted i-motif located upstream (at −371_−309) of the reference TSS, which represents a motif particularly well-conserved between human and mice. At the functional level, hnRNP-K binds to TARDBP promoter and appears to act as a dose-dependent activator of transcription in luciferase assays [170], possibly counterbalancing the action of TDP-43 in repressing promoter use [189,190].

In addition, the upstream promoter region of TARDBP appears to drive high transcriptional activity in cell lines of neuronal origin and in HeLa when compared to the non-neural human embryonic kidney 293 cells [173], suggesting that additional brain-specific transcription factors might bind to it. Moreover, specific factors might bind to and modulate TDP-43 promoter activity in favor of various stimuli. Notably, calcium-dependent neuronal hyperactivation, as induced by TEA, increases TDP-43 and isoforms transcription [159]. Moreover, the pro-inflammatory LPS stimulation of THD-1 macrophagic cells, provokes a transient increase in TDP-43 transcription [46]. In addition, it cannot also be discounted that additional regions of the bipartite promoter, which are specific to primates and poorly conserved in rodents [170,173], could contribute to a differential TARDBP regulation between these species.

In the 2009 study, non-coding DNA variants investigated in the upstream promoter or intron 1 region of the TARDBP gene were detected quite frequently (35%) in the brain (lateral frontal gyrus) of sALS patients (16/46), including two promoter variants found more frequently in sALS patients than in controls [172]. These detected SNPs did not modify the binding site of any known brain transcription factors nor affect promoter activity in several neuronal and non-neuronal cell lines (SH-SY5Y, Neuro2A, HeLa, HEK293), as tested recently [173]. However, it is quite possible that other mutations within TARDBP regulatory region, or alterations in factors that binds to it, could disrupt the regulation of TARDBP and be causative of the disease. Further work should be pointed in this direction.

A second potential line of regulation of TDP-43 transcription involves epigenetic modifications. Specifically, DNA methylation at CpGs has been found to be important for TDP-43 nonsense-mediated decay-isoform production and autoregulation, as reported above. In addition, CpG methylation is well known to regulate gene expression at promoter regulatory regions. The human TARDBP holds a large CpG island with at least three CpG clusters along upstream-intron1 bipartite promoter, accounting for 123 CpG [180,186]. In vitro manipulation of its methylation showed that changes in DNA methylation can profoundly affect promoter strength [186]. Specifically, a region directly upstream of the TSS holding 22 CpGs (CpG cluster2) was found to be largely hypomethylated (<2.5% at all CpGs) by bisulfite amplicon sequencing in three examined human brain areas (motor, occipital, and cerebellar cortices). Similarly, low levels were observed in the brains of healthy subjects and ALS patients [180], suggesting that changes in DNA methylation in this region may not be involved in ALS. Nonetheless, histones modifications or changes in DNA methylation at other regulatory regions, such as the intron1 (cluster 3) or in the most upstream region (cluster 1), which were found methylated in the human prefrontal cortex [180] and in SH-S5Y5 [186], have not being investigated yet, and could be of interest. Indeed, in the mouse, the predicted promoter holds a similar pattern of CpG island distribution [186], and an age-related increase in the methylation level of the CpG island in intron 1 has been found in brain and skeletal muscle tissues [186]. An increase in the repressive H3K27me3 and a decrease in the histone H2 variant H2Az deposition in this area and in the downstream gene body were also identified [186]. These are all epigenetic modifications, seemingly concurring to reduce the total *Tardbp* mRNA and TDP-43 expression as observed in normal aging mice in muscle and brain. It is therefore probable that similar epigenetic modifications modulate the expression of human TARDBP locus (although it will need to be tested). In addition, no such epigenetic changes were noted in the liver, suggesting some tissue-specific susceptibility [186].

## 4. Potential Druggable Targets

The treatment for ALS involves the multidisciplinary management of symptoms and the use of drugs, principally neuroprotective, neurotrophic, anti-inflammatory, antioxidative, and anti-glutamatergic drugs [191]. Many efforts have been made in finding a treatment for ALS, but none has shown a prolonged effectiveness. TDP-43 represents an attractive target, but due to its vital functions, ubiquitous expression and need for precise expression balance, delivery must be very precise in order to avoid being off target. From this perspective, the vector delivery of nucleic acids (NAs) such as ASOs, drugs, or mAbs against TDP-43 could constitute valuable strategies. NA therapeutics targeting some ALS-linked mutations are already under clinical investigation and have been recently reviewed [191,192]. Concerning mAb delivery, it was recently tested in vivo in transgenic mouse models of ALS/FFTD, showing mitigating effects such as cognitive impairment, motor defects, TDP-43 proteinopathy, and neuroinflammation [193]. The technology is centered on a vector-based delivery of a single-chain (scFv) anti-TDP-43 RRM1 antibody, the E6-antibody-derived VH7Vk9. Mechanistically, VH7Vk9 binding to TDP-43 enhanced the ubiquitination of the protein toward the proteasome and autophagic degradation pathways. The antibody also reduced microgliosis in a mouse model of acute neuroinflammation by blocking TDP-43 and NF-kB p65 and reducing NF-kB activation [193]. The same group tested the full-length antibody E6 in AAV-free delivery and found it to be internalized within cells, and even more efficient in targeting cytoplasmic TDP-43 to degradation, likely through TRIM21 and lysosome-dependent mechanisms and reduced NF-κB activation [194]. Importantly, E6 recognized, specifically, the cytoplasmic TDP-43. Diverse routes of delivery were tested, and repeated intrathecal injections for ALS appeared as the more therapeutically valid [194]. For a comprehensive review, see also the review by Poulin-Briere and colleagues [195].

Alongside TDP-43 itself, the many studies reported in this review also open routes to new targets for treatment, notably pharmacologic inhibition of retrotransposons, even in the absence of overt retrotransposition, or endogenous retroviruses proteins. In particular, inhibition of the HERV-K family env protein may mitigate the neurotoxic effects and/or spreading of TDP-43 pathology. The therapeutic use of an antiretroviral (ARV) cocktail has already been explored to some extent. Some HIV-infected patients develop ALS-like symptoms (HALS) [196], maybe linked to the presence of HIV DNA in the brain tissues [197], and also display HERV-K activation. A little group of such HALS patients were administrated ARV therapy and had either nearly complete motor recovery (if ARVs were administered within 6 months of symptom onset) or, at least, increased survival and an associated diminution in HERV-K products detected in blood [196]. The safety and tolerability of a long-term antiretroviral therapy (ART) using Triumeq (abacavir, lamivudine, and dolutegravir) has been recently tested in clinical trial phase 2 for HIV-negative ALS patients, and there have been suggestive indications of a possible biological response in some pharmacodynamic and clinical biomarkers, that ended particularly reduced [198]. Notably, a favorable response on HERV-K expression levels was observed, accompanied by a decline in ALSFRS-R progression rate of 21.8%. The clinical trial entered international phase 3 in February 2022 and will end in 2026 (ClinicalTrials.gov Identifier: NCT05193994). Here, it is important also to underline that, apart from the ARV effect on HERV-K, lamivudine is also a well-known inhibitor of the LINE-1 retrotransposition [199], widely used in research on L1’s physiopathological role, and thus may also counteract the negative effects of L1 activation discussed in this review.

HERV-K env glycoprotein, when produced in the diseased cells, should be directed to the cell plasma membrane and exposed at the surface. Indeed, focal accumulations of HERV-K env at the cell membrane of cortical neurons and motor neurons in the lumbar spinal cord have been evidenced in some ALS patients [141]. Thus, it could represent a more specific target for molecules such as mAbs. This option has already been envisaged for the treatment of some cancers in which HERV-K env is exploited as a tumor-specific antigen [200,201]. Another example is the Temelimab/GNbAC1, a humanized Ig4 monoclonal antibody against the protein from HERV-W/MSRV (multiple sclerosis-associated retrovirus) env protein associated with MS and siabetes type 1. MSRV is related to the HERV-W with, as yet, no perfectly matching sequences identified in the human genome, nor to a known exogenous retrovirus. It has the highest concordance (98%) with HERV-W X.q22.3b, and it is closely related to the domesticated HERV-W member ERVWE1 env glycoprotein Syncytin-1 on chromosome 7q21 [202]. Temelimab/GNbAC1 is currently in clinical development for the treatment of multiple sclerosis and type1 diabetes (ClinicalTrials.gov identifiers: NCT03574428 and NCT03179423) [202,203]).

Epigenetic modifications do not modify the DNA sequence but are generally stable. Many are reversible, and pathological TDP-43-related modifications, either caused by TDP-43 or causing TDP-43 alterations, could constitute a new era of therapeutic target investigations for TDP-43 proteinopathies. Targeted delivery of molecules modulating the toxic impact of TDP-43 alterations, such as the ones presented in the first paragraph on chromatin modifiers, could represent a path for new therapies and could, notably, benefit from novel pharmacological approaches derived from research against cancer [204].

Moreover, a more meticulous investigation on functions and targets specifically related to the short protein isoforms of TDP-43 on chromatin would bring further insight to the TDP-43/TARDBP pathologies and onset. Furthermore, it will bring a deeper understanding of TARDBP gene regulation and isoforms modulations, hopefully providing reliable future directions for new therapies. In particular, targeted manipulation of the DNA methylation status of the TARDBP 3′UTR could represent another way of correcting TDP-43 autoregulation. Technologies using dCas9-DNMT3a fusion protein constructs can direct specific on-site targeting of DNMT3a by accurately designing guide RNAs [205], or the homology-assisted repair-dependent epigenetic engineering (HARDEN) which uses an in vitro generated methylated repair template [206], could allow for stable targeted DNA methylation. The re-purposed CRISPR-Cas9 system, bringing either DNMT3a (methylating) or Tet1 (demethylating) proteins, was indeed used in the work of Koike et al. [180] on the impact of DNA on TDP-43 autoregulation. By using dCas9-Tet1, they were able to greatly demethylate TDP-43 CpG sites in the DNA region corresponding to the splicing regulation and impede the NMD-leading splicing, while dCas9-DNMT3a, on the contrary, correctly methylated this region [180]. HARDEN has been used to methylate the ALS-linked *C9orf72* repeat expansion in patients-derived iPSCs and the promoter of the APP protein involved in Alzheimer’s disease in HEK293T. The manipulation of DNA methylation notably employed HDR, not NEHJ [206]; thus, it should not be impacted by TDP-43 alterations, at least in cycling cells, and an AAV- and CRISPR–Cas9-mediated HDR has already been efficiently applied to post-mitotic neurons [207]. Although not yet ready as therapeutic targets, these tools definitely represent interesting means by which to understand the role of epigenetics in TDP-43-related disease and, more generally, in neurodegenerative diseases.

## 5. Concluding Remarks

In this review, we have emphasized the broad role of TDP-43 in the chromatin context, and, conversely, the potential role that could be played by the epigenetics on its own regulation. The results of all these studies show that TDP-43 (and potentially its shorter products) has many interconnections with chromatin and can profoundly impact chromatin homeostasis and gene expression through a range of different modalities. TDP-43 thus appears to be a pleiotrophic protein whose perturbation alters a wide variety of crucial cellular functions. 

It is important to state that most of the reported works have been performed on cellular models or on brain samples from ALS and FTD–ALS patients post-mortem. Therefore, we still lack confirmation in advanced disease models and in human early disease stages. Gaining information at earlier stages could presumably allow us to identify some targets as early biomarkers. In addition, as described in the introduction, ALS and FTD–ALS are not the unique neurodegenerative contexts in which TDP-43 abnormalities are observed. Notably, TDP-43 abnormalities in LATE, Alzheimer’s and Huntington’s diseases and brains after traumatic brain injury (TBI) have already been well described. However, TDP-43 inclusions are now shown to appear in a large panel of neurological and muscular disorders, as presented in a recent review [14]. Therefore, the causes and timing of TDP-43 alterations and their consequences in all these different pathologies are yet to be explored—in particular, those related to chromatin, along with the mechanisms leading to TDP-43 inclusions. 

Attention must also be drawn to the diversity of the lncRNAs interacting with TDP-43. We reported a variety of them, which we discussed in relation to the process in which their functional interaction with TDP-43 has been shown. However, we limited our review to the processes with a direct impact on chromatin: either chromatin eviction or targeting for transcriptional regulation, or else for DNA repair. Still, it is worth underlining that TDP-43 interaction with at least two other lncRNAs has been described, namely. NEAT1 long isoform (NEAT1_2) and LCETRL3. NEAT1 promotes TDP-43 inclusion in NEAT1 nuclear paraspeckles by liquid–liquid phase separation in case of stress, forming protective nuclear bodies [208]. The protective role of NEAT1 against TDP-43 toxicity has been attributed to a sponge-like action and proposed to bind and neutralize the excess of TDP-43 [209]. The role of NEAT1 in the regulation of TDP-43 and the roles of this particular interaction in neurodegenerative diseases have been recently and specifically reviewed [210]. It is so far not known if the sequestration of TDP-43 by NEAT1 has an impact on its localization, abundance, and function at chromatin. LCETRL3, standing for long cancer EGFR-TKI-resistant LncRNA 3, is a lncRNA that controls TDP-43 degradation in non-small-cell lung cancer by preventing its ubiquitination and proteasomal degradation [211]. New investigations would be needed in order to state whether this RNA, present also in the nucleus, is expressed in the brain and regulates TDP-43 load at chromatin.

An additional factor to be considered in TDP-43-linked pathologies is the role that gender has in the prognosis of ALS-FTD. Population-based datasets have highlighted a male prevalence of ALS cases with a 2:1 gender ratio [212], although more recent studies have reported a decrease in the ratio with a trend towards 1:1 [213]. Nowadays, the standardized male-to-female ratio is 1.35 [214], although a meta-analysis on genetic mutations linked to FTD revealed a higher prevalence of female patients with the C9-related ALS/FTD [215]. A study performed on the TDP-43^Q331K^ knock-in mouse reported weight gain and age-dependent increases in food intake and a generally intact innate exploratory digging behavior in the mutant female compare to the male [216]. Thus, future investigations on the influence that TDP-43 regulation/functions exercises in relation to sex/gender, environment, lifestyle, or diet would surely bring relevant information.

Finally, the new technological possibilities enabling single-cell analysis or TDP-43 nuclear loss-based differential sorting appear crucial. Such cell partitioning can be used for single or multi-omic data analysis, for example, such as in transcription, DNA methylation, or chromatin accessibility [217], to identify susceptible neuronal subtypes and represent a road towards an in-depth understanding TDP-43 proteinopathies.

## 6. Resources for the Review

Sources specific to the epigenetic aspects regarding TDP-43 for this review were identified via PubMed searches using the key terms “TDP-43”, “TDP43”, or “TARDBP” AND the epigenetic features “DNA methylation”, “Methylation ”, “Chromatin”, “Chromatin associated”, or “Epigenetics”, or the RNA/DNA elements “lncRNA ”, “miRNA”, “Retrotransposons”, or technical methods “ChIP”, “RIP”, “RNA-IP”, or “ATAC-seq”. No time limitation was applied (up to July 2023), with the aim of covering the thematic of the review as exhaustively as possible, including its implication for neurodegenerative pathologies. Additional sources were taken from the references used in the above-retained research or review articles and from the publications already known by the authors. No bias was assigned for authors or institutions. Manual curation was performed to remove protein post-translational modifications (e.g., methylation and acetylation) not directly linked to TDP-43 epigenetic regulation. Likewise, resources treating the implication of TDP-43 in RNA splicing with no clear consequences for epigenetic processes or in miRNA processing were not included as they were already covered by other recent reviews. When a review was used as a resource, it was cited in the text. 

## Figures and Tables

**Table 1:** Chromatin and transcription factors directly or indirectly regulated by TDP-43 and modifying TDP-43-induced toxicity.

Gene Symbol	Name	Mammalian Orthologs *, Alias	Gene Ontology Function	Contribution to Chromatin Structure/Transcription	Toxicity Modulation upon Knockdown	Refs.
*Chd1* ^†^	*Chromodomain-helicase-DNA binding protein 1*	*CHD1, CHD2*	Chromatin remodeling	SILENCING	suppresses/ameliorates	[52]
*e(y)3* ^†^	*Enhancer of yellow 3*	*PHF10*	Chromatin remodeling	OPENING	suppresses/ameliorates	[52]
*polybromo* ^†^	*Polybromo*	*BAF180*	Chromatin remodeling	OPENING	suppresses/ameliorates	[52]
*ash1* ^†^	*Absent, small, or homeotic discs 1*	*ASH1L*	Chromatin remodeling	OPENING	suppresses/ameliorates	[52]
*enok* ^†^	*Enoki mushroom*	*KAT6A*	Chromatin remodeling	OPENING	suppresses/ameliorates	[52]
*br* ^†^	*Broad*	*-*	Chromatin remodeling	OPENING	suppresses/ameliorates	[52]
*Br140* ^†^	*Bromodomain-containing protein, 140kD*	*BRPF1*	Chromatin remodeling	OPENING	suppresses/ameliorates	[52]
*mor* ^†^	*Moira*	*BAF170S/MARCC2*	Chromatin remodeling	OPENING	suppresses/ameliorates	[52]
*MED8* ^†^	*Mediator complex subunit 8*	*MED8*	Mediator complex	TF–RNA Pol interaction	suppresses/ameliorates	[52]
*MED11* ^†^	*Mediator complex subunit 11*	*MED11*	Mediator complex	TF–RNA Pol interaction	suppresses/ameliorates	[52]
*MED15* ^†^	*Mediator complex subunit 15*	*MED15*	Mediator complex	TF–RNA Pol interaction	suppresses/ameliorates	[52]
*MED21* ^†^	*Mediator complex subunit 21*	*MED21*	Mediator complex	TF–RNA Pol interaction	suppresses/ameliorates	[52]
*MED22* ^†^	*Mediator complex subunit 22*	*MED22*	Mediator complex	TF–RNA Pol interaction	suppresses/ameliorates	[52]
*MED27* ^†^	*Mediator complex subunit 27*	*MED27*	Mediator complex	TF–RNA Pol interaction	suppresses/ameliorates	[52]
*MED28* ^†^	*Mediator complex subunit 28*	*MED28*	Mediator complex	TF–RNA Pol interaction	suppresses/ameliorates	[52]
*MED31* ^†^	*Mediator complex subunit 31*	*MED31*	Mediator complex	TF–RNA Pol interaction	suppresses/ameliorates	[52]
*e(y)1* ^†^	*Enhancer of yellow 1*	*TAF9*	Transcription Factor	Transcription	suppresses/ameliorates	[52]
*TAF1* ^†^	*TBP-associated factor 1*	*TAF1*	Transcription Factor	Transcription	suppresses/ameliorates	[52]
*Tomb* ^†^	*tombola*	LIN54/TESMIN **	Male meiosis	Transcription	suppresses/ameliorates	[52]
*Su(Tpl)* ^†^	*Suppressor of Triplolethal*	*ELL, ELL2*	Elongator	Transcription	suppresses/ameliorates	[52]
*SF2* ^†^	*Splicing factor 2*	*SRSF1*	Splicing	Transcription	suppresses/ameliorates	[52]
*Hsp70B (Ba/Bb/Bbb/Bc)* ^†^	*Heat-shock-protein-70B (Ba/Bb/Bbb/Bc)*	*HSPA1A*	Heat shock Factor	Transcription	suppresses/ameliorates	[52]
*Chd1* ^†^	*Chromodomain-helicase-DNA-binding protein 1*	*CHD1*	Chromatin remodeling	TDP-1/TDP-43 impedes Chd1 binding	strongly enhances	[51]
*dom* ^†^	*Domino*	*SRCAP* **	Chromatin remodeling		strongly enhances	[51]
*mi-2* ^†^	*Mi-2*	*CHD3; CHD4; CHD5* **	Chromatin remodeling		enhances	[51]
*kis* ^†^	*Kismet*	CHD6, CHD7, CHD8, CHD9 **	Chromatin remodeling		enhances	[51]
*asf1* ^†^	*Anti-silencing factor 1*	*ASF1A; ASF1B*	Chromatin remodeling		suppresses	[51]
*Snr1* ^†^	*Snf5-related 1*	*SMARCB1*	Chromatin remodeling		suppresses	[51]
*mor* ^†^	*Moira*	SMARRC1; SMARCC2 **	Chromatin remodeling		suppresses	[51]
*Dalao* ^†^	*Brahma associated protein 111kD, Bap111*	SMARCE1 **	Chromatin remodeling		suppresses	[51]
*Chd3* ^†^	*Chd3*	*CHD3*	Chromatin remodeling		suppresses	[51]
*Bap60* ^†^	*Brahma associated protein 60kD*	*SMARCD1*	Chromatin remodeling		strongly suppresses	[51]
*MBD-R2* ^†^	*MBD-R2*	*PHF20; PHF20L1*	Histone Lysine acetyltransferase	OPENING	enhances	[51]
*Tip60* ^†^	*Tat interactive protein 60kDa*	KAT5; KAT7; KAT8 **	Histone Lysine acetyltransferase	OPENING	enhances	[51]
*CG2051* ^†^	*Histone acetyltransferase 1*	*HAT1*	Histone Lysine acetyltransferase	OPENING	suppresses	[51]
*HDAC3* ^†^	*Histone deacetylase 3*	*HDAC3*	Histone Lysine deacetylase	SILENCING	enhances	[51]
*Mta1-like* ^†^	*Metastasis associated 1-like*	*MTA2; MTA3*	Histone Lysine deacetylase	SILENCING	enhances	[51]
*Sirt4* ^†^	*Sirtuin 4*	*SIRT4*	Histone Lysine deacetylase	SILENCING	suppresses	[51]
*HDAC6* ^†^	*Histone deacetylase 6*	*HDAC6*	Histone Lysine deacetylase	SILENCING	suppresses *	[51]
*Scm* ^†^	*Sex comb on midleg*	*L3MBTL3; PHC2; SCML1; THAP10*	Transcription Factor	Transcription	suppresses	[51]
*Spt6* ^†^	*Spt6*	*SUPT6H*	Transcription Factor	Transcription	suppresses	[51]
*lid* ^†^	*Little imaginal discs*	*KDM 4A; 4B; 4D; 4E; 4F; 5A; 5B; 5C; 5D;*	Histone Lysine demethylase (H3K4)	SILENCING	strongly suppresses	[51]
*Utx* ^†^	*Utx histone demethylase*	*KDM6A; 6B; UTY*	Histone Lysine demethylase (H3K27)	OPENING	suppresses	[51]
*Asx* ^†^	*Additional sex combs*	*ASXL1-3*	Chromatin regulator	SILENCING	suppresses	[51]
*Ncoa6* ^†^	*Nuclear receptor coactivator 6*	*NCOA6*	co-activator	Transcription	suppresses	[51]
*Su(var)3-9* ^†^	*Suppressor of variegation 3-9*	*SUV39H1*	Histone Lysine methyltransferase (H3K9)	SILENCING	suppresses	[51]
*ash2* ^†^	*Absent, small, or homeotic discs 2*	*ASH2L*	Histone Lysine methyltransferase (H3K4)	OPENING	enhances	[51]
*Su(z)2* ^†^	*Suppressor of zeste 2*	*BMI1; PCGF1; 2; 5; 6; RING1B; RNF2*	Histone Lysine methyltransferase, histone monoubiquitination (H2A-K119)	OPENING	enhances	[51]
*Set1* ^†^	*SET domain containing 1*	*KMT2B*	Histone Lysine methyltransferase (H3K4)	OPENING	enhances	[51]
*Wdr82* ^†^	*WD repeat domain 82*	*WDR82*	Histone Lysine methyltransferase (H3K4)	OPENING	enhances	[51]
*HPL-2* ^†2^	*Heterochromatin Protein-like 2*	*HPL1*	Chromatin remodeling	TDP-1 bridges HPL-2 to chromatin	n.d.	[53]
*nBAF complex*	*Neuronal Brg1/BRM Associated factor*	*SS18L1, NBAF; CREST; SYT homolog 1*	Neuronal chromatin remodeling complex nBAF component		n.d.	[57]
*sgg* ^†^	*Shaggy*	*GSK3*	Control of cellular pathways and metabolism		suppresses	[50]
*htk* ^†^	*Hat-trick*		Chromatin remodeling		suppresses	[50]
*xmas-2* ^†^	*Xmas-2*		Transcription and RNA export		suppresses	[50]
*H3C14*	*H3S10Ph-K14Ac: Histone 3 (serine 10 phosphorylation/lysine 14 acetylation)*	*H3*	Histone tail PTM	Transcriptional activation/repression	n.d.	[58]
*H3C14*	*H3K9me3: Histone 3 (methylation of lysine 9)*	*H3*	Histone tail PTM	Transcriptional repression	n.d.	[58]
*HDAC1*	*Histone deacetylase 1*	*HDAC1*	Histone/Protein Lysine deacetylase	SILENCING ***	n.d.	[48]
*H2b*	*Histone cluster 1, H2bp*	*HIST1H2BP*	Nucleosome assembly	Downregulated in the TDPΔNLS	n.d.	[63]
*H3d*	*Histone cluster 1, H3d*	*HIST1H3D*	Nucleosome assembly	Downregulated in the TDPΔNLS	n.d.	[63]
*H4a/H4b/H4c/H4h*	*Histone cluster 1, H4a, b, c, h*	*HIST1H4*	Nucleosome assembly	Downregulated in the TDPΔNLS	n.d.	[63]
*Nap1l1*	*Nucleosome assembly protein 1-like1*	*NAP1L1*	Nucleosome assembly	Upregulated in the TDPΔNLS	n.d.	[63]
*Med20*	*Mediator Complex subunit 20*	*MED20; SRB2; TRFP; PRO0213*	Mediator Complex/transcriptional coactivator	Transcriptional activation	n.d.	[64]
*Usp49*	*Ubiquitin specific processing protease 49*	*USP49*	H2B Histone deubiquitinase	Transcriptional activation/regulation of mRNA splicing	n.d.	[64]
*HUWE1*	*HECT, UBA, WWE domain Containing E3 Ubiquitin Protein ligase 1*	*HUWE1*	Histone/protein Ubiquitination		n.d.	[65]
*YY1*	*Yin and Yang 1 transcription Factor*	*YY-1; INO80S*	Transcription factor	Transcriptional activation/repression	n.d.	[65]
*MORF4L2*	*Mortality Factor 4-like 2*	*MRGX; KIAA0026; MORF-Related Gene X*	Heterochromatin assembly/histone modification	Transcriptional activation	n.d.	[65]
*HMGN1*	*High-Mobility Group Nucleosome Binding Domain 1*	*HMG14; MGC104230; FLJ27265*	Chromatin remodeling		n.d.	[65]
*PRKDC*	*Protein Kinase, DNA-Activated, Catalytic subunit*	*DNA-PKcs; DNPK1; P460; DNAPKc; XRCC7; P350*	DNA repair and recombination		n.d.	[65]
*UIMC1*	*Ubiquitin Interaction Motif Containing 1*	*RAP80; Retinoid X Receptor-Interacting Protein 110*	DNA repair	Ubiquitination/Transcription repression	n.d.	[65]
*POLB*	*DNA Pol Beta*	*DNA Pol Beta*	DNA excision and repair		n.d.	[65]
*SFPQ*	*Splicing Factor Proline and Glutamine Rich*	*PSF; PPP1R140; HPOMp100; POMP100*	Splicing	Enables DNA binding activity	n.d.	[65]
*MSH3*	*MutS Homolog 3*	*MRP1; DUP; HMSH3; Mismatch Repair Protein*	DNA mismatch repair system (MMR)	Postreplicative DNA mismatch repair	n.d.	[65]
*XRCC5*	*X-Ray Repair Cross Complementing 5*	*Ku86; KARP-1; KU80; KUB2*	DNA repair	DNA DSB repair by NHEJ	n.d.	[65]
*XRCC6*	*X-RAY Repair Cross Complementing 6*	*KU70; Cells6; G22P1; ML8*	DNA repair	DNA DSB repair by NHEJ	n.d.	[65]
*STAG2*	*Stromal Antigen 2*	*SA2; SCC3B; SA-2; Cohesin Subunit SA-2*	Chromatin remodeling	Cohesion of sister chromatids after DNA replication	n.d.	[77,78]
*Ell, Su(Tpl)* ^†^	*Elongation Factor for RNA Pol II*	*ELL; ELL2; C19orf17; PPP1R68*	Elongator	Transcription regulation	enhances	[52,79]

^†^ Flybase, ^†2^ Wormbase * Mammalian Orthologs from referenced work or from OrthoDB database. ** Contribution to chromatin structure/transcription. Source: referenced work, OrthoDB, NCBI, or UniProt databases. *** Potentially by acting directly on TDP-43 acetylation. Histone PTM stands for histone post-transcriptional modification.

**Table 2:** Epigenetic landscape modifications and functional impact on chromatin of ALS TDP-43 mutations.

TDP-43 Mutation		Epigenomic Alterations	Cellular Context	Refs.
G294V	DNA damage/DSB TC-DSB	Mislocalization;	SH-S5Y5	[97]
		R-loop accumulation; R-loop-dependent increased DSBs		
		Accumulation of FANCD2 repair foci (replication blockage).		
G298S	DNA methylation	Hyper- and hypo-methylated regions related to controls with common and specific DMRs related to other ALS hIPSCs-derived MNs (C9orf72-, TARDBP-, SOD1-, and FUS-mutation carriers).	hIPSCs-derived MNs from G298S carrier	[74]
A315T	Chromatin remodeling	Diminution of nBAF chromatin-remodeling complex components (Brg1, BAF53b, and CREST).	Cultured mouse MNs	[57]
	DNA methylation	Altered 5mC and 5hmC underlying their increased vulnerability to degeneration.	TDP-43^A315T^ mouse CSMNs	[171]
	DNA damage/DSB	Enhanced vulnerability to DNA damages.	MN-like NSC-34 cells	[95]
M323K	Autoregulation	Increased RNA, but not significant for protein.	Mouse TDP-43^M323K^ knock-in	[167]
Q331K	Autoregulation	Increase in RNA and nuclear protein in frontal cortex and spinal cords of mutant mice, but not in motor neuron.	Mouse TDP-43^Q331K^ knock-in but not transgene	[168,169]
	DNA damage/DSB	Increased cytosolic sequestration of the poly-ubiquitinated and aggregated form, nuclear loss of function DNA damage induction, and DSB repair defects (preventing the nuclear translocation of XRCC4); Contribution to oxidative genome damage accumulation via increased reactive oxygen species (ROS).	Spinal cord of ALS Q331K carriers, SH-S5Y5	[93]
		Enhanced vulnerability to DNA damages (increase yH2Ax post damaging agent exposure).	MN-like NSC-34 cells	[95]
M337V	DNA methylation	No global alteration in 5mC	SH-S5Y5	[58]
	Histone PTMs	Significant decrease in global H3S10Ph-K14Ac; No significant increase in H3K9me3.		
	DNA damage/DSB	Impairment in the NHEJ DSB repair factors recruitment.	Fibroblasts of human M337V carrier	[95]
G348C	Autoregulation	Upstream intron1 promoter region of TARDBP binding but less efficient than WT for its repression; No significant activation of the intron 1 promoter.	HEK293T	[170]
A382T	DNA Methylation	No global alteration in 5mC.	SH-S5Y5	[58]
	Histone PTMs	No significant decrease in global H3S10Ph-K14Ac; No significant increase in H3K9me3.		
	DNA damage/DSB TC-DSB	Increased yH2Ax; R-loop accumulation; R-loop-dependent increased DSBs accumulation of FANCD2 repair foci (replication blockage).	SH-S5Y5 LCL from A382T carriers	[97]
	Isoforms/fragments	Increased TDP-35 form at chromatin at R-loop.	LCL from A382T carriers	[97]
	Autoregulation	Upstream intron1 promoter region of TARDBP binding but less efficient than WT for its repression; Able to induce a significant activation of the intron 1 promoter.	HEK293T	[170]
N390D	DNA methylation	Hyper- and hypo-methylated regions related to controls with common and specific DMRs related to other ALS hIPSCs-derived MNs (C9orf72-, TARDBP-, SOD1-, and FUS-mutation carriers).	hIPSCs-derived MNs from N390D carrier	[74]
G418C	Chromatin remodeling	Diminution in nBAF chromatin-remodeling complex components (Brg1, BAF53b and CREST).	Cultured mouse MNs	[57]
C9orf72 with loss of nuclear TDP-43	DNA methylation	Altered DNA 5mC and 5hmC in the CSMN without nuclear TDP-43 compared to residual C9orf72 CMSN without TDP-43 nuclear loss.	Residual lower MNs from post-mortem CSMN tissues of C9orf72 carriers	[73]

MNs: motor neurons; CSMNs: corticospinal MNs; LCL: lymphoblastoid cell line.

## Data Availability

Not applicable.
